# Unpacking mating success and testing Bateman’s principles in a human population

**DOI:** 10.1098/rspb.2019.1516

**Published:** 2019-08-14

**Authors:** Monique Borgerhoff Mulder, Cody T. Ross

**Affiliations:** 1 University of California, Davis, Department of Anthropology, Davis, CA, USA; 2 Max Planck Institute for Evolutionary Anthropology, Department of Human Behavior, Ecology and Culture, Leipzig, Germany

**Keywords:** polygyny, serial monogamy, polyandry, evolutionary demography, sexual selection

## Abstract

Human marriage systems, characterized by long-term partnerships and extended windows of parental care, differ from the mating systems of pulsed or seasonally breeding non-human animals in which Bateman’s principles were originally tested. These features, paradigmatic of but not unique to humans, complicate the accurate measurement of mating success in evaluating Bateman’s three principles. Here, we unpack the concept of mating success into distinct components: number of partners, number of years partnered, the timing of partnerships, and the quality of partners. Drawing on longitudinal records of marriage and reproduction collected in a natural-fertility East African population over a 20-year period, we test and compare various models of the relationship between mating success and reproductive success (RS), and show that an accurate assessment of male and female reproductive behaviour requires consideration of all major components of mating success. Furthermore, we demonstrate that while Bateman’s third principle holds when mating success is defined in terms of years married, women’s fitness increases whereas men’s fitness decreases from an increase in the number of marriage partners, holding constant the total effective duration of marriages. We discuss these findings in terms of the distinct, sex-specific pathways through which RS can be optimized, and comment on the contribution of this approach to the broader study of sexual selection.

## 1. Introduction

### (a) Bateman’s principles and human marriage systems

As observed in most other mammalian populations [1], human groups are typically characterized by greater variance in male than female reproductive and mating success (Bateman’s 1st and 2nd principles [2]), and stronger effects of mating success on reproductive success (RS) in males relative to females (Bateman’s 3rd principle) [3–5]. However, existing approaches to evaluating Bateman’s principles in humans have applied models best suited to non-human animals that often have very different mating systems. Here, we explore how testing Bateman’s principles in humans—a species characterized by pairbonding and extended periods of parental care—using annually resolved marriage history data can shed light on hitherto under-appreciated aspects of male and female reproductive strategies. We use new data from a natural-fertility population and a finely resolved set of measures of mating success to both support Bateman’s principles and document a previously unrecorded pattern in which women’s RS increases and men’s RS decreases from an increase in number of marriage partners, holding constant the total effective duration of marriages (i.e. summed over all partners).

Studies of Bateman's principles in non-humans differ in the specific components of mating success that are measured, namely: mating frequency, number of fertilizations, number of mates and/or mate quality [6–8]. Which measures are used depends on whether behavioural, demographic and/or genetic data are most readily available, on the likelihood that matings lead to RS, and on the possibility that post-mating competition influences RS. Studies of Bateman’s principles in humans, by contrast, generally use number of marriage partners (e.g. [3,5,9–12]), with the exception of one recent analysis [13]—but see [14]—that measures the proportion of adult lifespan spent married. Insofar as sexual selection might select for acquiring more mates, copulating more frequently at appropriate times, pairing for longer durations, and/or choosing higher-quality mates, non-human studies are beginning to unpack these components of mating success [15] and look at their potential trade-offs [16]. The same level of resolution should be applied to human case studies.

We propose that in pairbonding species, and in species with extended parental care, both the total number of years an individual is partnered and the total number of partners can provide distinct information about mating success. The former—a measure of the duration of reproductive access—approximates the concept of mating success as number of matings; the latter provides additional information with regard to the sex-specific strategies on which sexual selection might operate. In humans, a greater number of spouses might increase social and affinal kin network connections, resource access, and offspring survival [17]; likewise, in some non-human mammals, a greater number of mates might reduce infanticide risk by males via the confusion of paternity [18]. Accordingly, we disentangle the distinct effects of each measure for each sex.

Furthermore, not all years partnered to a mate are of equal value to reproduction. Failing to account for both the age at which a focal individual is partnered and the age of his or her partner can lead to misestimation of variation in mating success and Bateman gradients (the effects of mating success on RS). To rectify this, we weight the years in which an individual is partnered with value functions defined over the age of both the focal individual and his/her partner in a given year, capturing more cleanly mating success over the time-frames where variance in mating success is relevant to RS. This approach allows us to move beyond simple contrasts of the benefits of multiple mating for males and females, and consider *the paths through which* multiple mating affects RS, paralleling recent approaches in non-human studies [15,16]. We draw methodological inspiration from the empirical literature on Bateman gradients, but we extend extant path analytic and variance decomposition approaches by using custom-built Bayesian models to avoid some common, but biologically implausible, modelling assumptions. Our models: (i) represent the data-generating process, (ii) account for zero-inflated outcomes, (iii) account for diminishing marginal returns to RS inputs, and (iv) estimate the unknown value functions linking expected reproductive output and the ages of focal individuals and their spouses in each year of marriage.

Systematic studies that compare how mating success affects the RS of men and women are rare. To date, such investigations have been conducted primarily in populations practicing serial monogamy, where women cannot typically initiate divorce, and where remarriage is driven by widowhood [5,19,20], raising concerns over the endogeneity of marital ‘decisions’ (e.g. [19]). While populations exist where females do have agency—e.g. in societies with ‘informal polyandry’ [21] and societies where biological paternity is believed to be shared among the recent sexual partners of a given woman [17]—individual-level data revealing the RS consequences of variation in mating success therein are not available. To address these limitations, we conducted a longitudinal demographic study of the Pimbwe of western Tanzania, where cultural norms allow both men and women to marry, divorce, and remarry with largely free agency [10].

### (b) Unpacking mating success

Under some conditions, the use of number of mates as a proxy for overall mating/copulation success is not problematic; for example, in pulsed or seasonally breeding species, number of mates may be an acceptable measure of an individual's total reproductive access. In pairbonding species with long periods of bi-parental investment, however, it may be important to disentangle the effects of number of mates on RS from the effects of other factors, like the total number of years partnered. In such groups, RS may depend on the number of acquired mates, but also on the time spent partnered to each, their quality, and the extent of their investment in offspring.

To operationalize mating success in humans, we supplement the commonly used measure *spouse number* with the additional measure *marital years* [13]. While we recognize that measures of marriage and mating are not substitutes, we propose that our approach of unpacking the distinct effects of number of mates and duration of partnerships on RS can be applied in species where mating pairs are formed and where within-population variation in the duration of such partnerships contributes to mating competition. For clarity, from here on, we switch to human-specific terminology, using, for example, *marriage success* instead of *mating success*.

Marital years are calculated as the total number of years an individual is married, counting the independent contributions of concurrent spouses (e.g. in the case of a polygnyous man with more than one wife). A greater number of years spent in marriage should result in higher fitness, for men primarily because they have longer and more consistent sexual access and for women primarily because they gain longer and more consistent provisioning. Holding constant the effects of marital years, elevated spouse number might proxy increased genetic variability in offspring, higher spousal quality (if lower quality spouses are replaced with better ones), as well as increased social network connections (effectively, a greater number of individuals as possible providers). In marriage systems characterized by both concurrent polygyny and serial monogamy, different individuals may have the same number of total spouses and yet a very different number of years married to these spouses, and hence different levels of total *exposure time* to fertile partners. For this reason, the use of spouse number as a single measure of mating success can be problematic. The distinct effects of number of mates and number of matings on RS have been identified in non-humans [22,23] and the parallel distinction in humans noted as potentially important [24].

While marital years may serve as a reasonable proxy for mating success in humans, not all years of life are of equal value to the production of offspring. In many societies, marriages extend into both pre- and post-reproductive periods, and variance in marriage success during these life-phases is unlikely to contribute as much to variance in RS as variance in marriage success during the reproductive life-phase. Accordingly, we recognize that the contribution of a marital year to our metric of marriage success should—minimally—account for some function of both the focal individual’s age, and the age of his or her spouse(s). Specifically, we estimate the number of *effective marital years* of an individual by adjusting the contribution of each year of marriage with weighting functions that are estimated endogenously within the model using Gaussian random fields; this approach allows for estimation of the relationship between age and expected reproductive output without imposing a specific functional form. In future work, these weighting functions can be extended to account for other factors that influence reproduction (e.g. health status or material wealth), as recommended by Henshaw *et al.* [15].

### (c) Research goals

To better understand the extent of variation in, and the relationship between, marriage success and RS in the Pimbwe, we revisit a previous analysis [10], this time unpacking the influence of each component of marriage success, and using a larger number of individuals, sampled over a longer period of time. After testing Bateman’s first principle, we examine his second and third principles using different measures of mating success: the number of spouses ever married (spouse number), the total number of years married (marital years), and then marital years accounting for marriage timing weights, spousal age weights, and both weights together. Finally, using a unified model, we test if there are sex differences in the pathways through which men and women maximize RS (see the electronic supplementary material, figure S10 for a schematic representation of the full model). Specifically, we ask, holding constant spouse number, are there sex differences in the effects of effective marital years on RS?, and, holding constant effective marital years, are there sex differences in the effects of spouse number on RS?

## 2. Background and methods

### (a) Ethnography

The modern administrative area of Mpimbwe is settled primarily by the Pimbwe and related Bantu groups [25]. As erstwhile residents of what is now Katavi National Park, the Pimbwe have a history of extensive hunting and fishing [26] that has become increasingly tenuous under twenty-first century conservation policies. Both men and women cultivate cassava and maize. Yields are unreliable owing to unpredictable rainfall, soil depletion, crop pests and theft. Men supplement farming with hunting, fishing and honey production; both men and women engage in off-farm activities like beer brewing, traditional medicine, and petty trade [27]. The area has been poorly served administratively and in terms of infrastructure for the last 100 years [26].

Serial monogamy with limited polygyny is predominant, with many men and women marrying multiple times [10]. Marriages are recognized when a couple decides to cohabit; likewise, reproductive partnerships are almost always acknowledged as marriages. Writing in the 1930s, Père Maurice [28, p. 189] (perhaps with missionary zeal) notes that traditional marriage in Mpimbwe is a ‘feeble institution’. Contemporary marriage entails coresidence, an expectation of sexual fidelity, shared provisioning and use of household product and labour, and obligations of respect to in-laws. In the case of divorce, unweaned offspring stay with their mothers; after weaning, they may live with either parent, often drifting between households.

We define marriage here, following the Pimbwe, as a recognized coresidence of sexual partners, acknowledging that spouse number, even in this highly behavioural definition, will probably underestimate the actual number of lifetime sexual partners. Ethnographic research reveals that some couples stay partnered for life; others marry and remarry sequentially (cf. Père Maurice’s ‘fragile contract’ [28, p. 189]), with either men or women taking the lead on divorce actions. Divorces are often associated with spousal violence, intra-household theft, or other disturbances, such as extramarital affairs, that typically lead to new marriages. Interviews indicate that both sexes exploit the flexible norms allowing monogamy, serial polygyny, serial polyandry, and concurrent polygyny to negotiate reproduction in an ecology with little infrastructure, poor food security, high disease burdens, and considerable material inequality.

### (b) Data collection

Demographic data (on births, deaths, marriages and divorces) were collected at all households in the village of Mirumba over a period of 20 years in seven full censuses (1995–2010) and two incomplete censuses (2012, 2014). For this analysis, all individuals older than 11 (the earliest age of parenting) with complete records were included (*n* = 1713). RS, a population-specific proxy for fitness, is defined in this analysis as the number of offspring surviving to 5 years. See the electronic supplementary material, S1.1 for details.

### (c) Statistical methods

To make our work comparable to recent meta-analyses [1], we report the standardized variance in RS (known as the ‘opportunity for selection,’ *I*) and the standardized variance in mating success (the ‘opportunity for sexual selection,’ *I*_*s*_) among the subset of individuals of age 45 or older. We use post-reproductive individuals for these measures, because variation in RS and marriage success depends heavily on age, which we can hold effectively constant by using only older individuals. Because the reproductive window can be longer in males than females, we also replicate our analysis among the subset of individuals of age 55 or older, but the sample here is smaller and might under-represent the extent of male reproductive inequality owing to secular increases in the frequency of polygyny (see the electronic supplementary material, S4.1.1).

To calculate the Bateman gradient, we use the full sample of individuals. Because our data show signs of non-linearity (diminishing marginal returns to marriage success), we measure the Bateman gradient using an *elasticity* parameter, which indicates the per cent change in RS with respect to the per cent change in marriage success. This is a standard approach in economics to modelling reproduction and marriage [29] when diminishing marginal returns to marriage success occur. Standard slope coefficients, however, can be calculated from our parameter estimates (see the electronic supplementary material, S2.1).

To appropriately model the generative process of our zero-inflated RS data (see the electronic supplementary material, S2.2), we use a two-stage modelling framework [30]. First, we model a binary indicator representing if individual, *i*, has at least a single year of marriage, *M*_[*i*]_, as a function of age:

2.1 $$M_{\left[i\right]}∼\;\text{Bernoulli}(\text{logistic}(\alpha_{\left[1,S(i)\right]}+\alpha_{\left[2,S(i)\right]}\log(E_{\left[i\right]}))),$$

where *E*_[*i*]_ is the *exposure time* to the possibility of reproduction—i.e. years lived in the interval between age 11 and death/censoring—and *S*(*i*) is a function returning an indicator for the sex of individual *i*.

In cases where *M*_[*i*]_ = 0, we expect that the RS, *R*_[*i*]_, of individual, *i*, is equal to zero as well, given the nature of sexual reproduction. Cases of *M*_[*i*]_ = 0, and the corresponding cases of *R*_[*i*]_ = 0, are tightly linked to age, with the probability that *M*_[*i*]_ = 0 itself approaching zero for individuals over age 30 of either sex (see the electronic supplementary material, S3.2 for a full treatment of the results of this submodel, and electronic supplementary material, S3.3 for evidence justifying the zero-inflated modelling approach).

In cases where *M*_[*i*]_ = 1, we fit our main model linking marriage success and RS. Specifically, we model *R*_[*i*]_ of individual, *i*, using a negative binomial outcome distribution [29]:

2.2 $$R_{\left[i\right]}∼\;\text{negative binomial}(\mu_{\left[i\right]}B_{\left[S(i)\right]},B_{\left[S(i)\right]}),$$

where the term *μ*_[*i*]_*B*_[*S*(*i*)]_ defines the shape parameter of a Gamma distribution, and *B*_[*S*(*i*)]_ defines the inverse scale parameter. This is equivalent to using a Gamma–Poisson mixture model, as has been recommended for modelling *over-dispersed* fertility-related outcomes—i.e. where the variance exceeds the mean—which are commonly found in polygynyous societies [31]. We can then define a model of mean RS, *μ*_[*i*]_, using a standard log link function:

2.3 $$\log(\mu_{\left[i\right]})=\beta_{\left[1,S(i)\right]}+\beta_{\left[2,S(i)\right]}\log(E_{\left[i\right]})+\beta_{\left[3,S(i)\right]}\log(N_{\left[i\right]})+\exp(\beta_{\left[4,S(i)\right]})\log(Y_{\left[i\right]}),$$

where the new variables are: spouse number, *N*_[*i*]_, and effective marital years, *Y*_[*i*]_.

*Y*_[*i*]_ for an individual is obtained by calculating a weighted sum of the number of years in which he or she has been married to each of his or her spouses:

2.4 $$Y_{[i]}=\overset{E_{[i]}}{\underset{e=1}{\sum }}\overset{N_{[i,e]}}{\underset{n=0}{\sum }}\{\begin{array}{cc} 0, & \mathrm{if}\;n\;=\;0\\ \underset{\begin{array}{c} \mathrm{value}\;\mathrm{to}\;a\;\mathrm{focal}\;\mathrm{individual}\;\mathrm{of}\;a\\ \mathrm{marital}\;\mathrm{year}\;at\;a\;\mathrm{given}\;\mathrm{age} \end{array}}{\underbrace{\mathrm{logistic}(\theta_{[S(i)]}+\nu_{[e,S(i)]})}}\;\cdot \underset{\begin{array}{c} \mathrm{value}\;\mathrm{to}\;a\;\mathrm{focal}\;\mathrm{individual}\;\mathrm{of}\;\mathrm{marriage}\\ \mathrm{to}\;\mathrm{spouse}\;of\;\text{a given age} \end{array}}{\underbrace{\mathrm{logistic}(\phi_{[S(i)]}+\psi_{[A(n,i,e),S(i)]}),}} & \mathrm{if}\;n\;>0, \end{array}\;$$

where the first factor gives the estimated value to the focal individual of having a spouse *at* a given age (a marriage timing weight) and the second factor gives the estimated value to the focal individual of having a spouse *of* a given age (a spousal quality weight). We note that effective marital years and spouse number are not strongly correlated (for men *ρ* = 0.3; for women *ρ* = 0.05). For further descriptions of model parameters, details about the hierarchical model structure, priors, software, and model fit diagnostics, see the electronic supplementary material, S2.2.1, S2.2.2, S2.2.3, S2.2.4 and S3.1, respectively.

We conduct several robustness checks of our analysis. First, we use an older age threshold of 55 years for the variance measures. Additionally, we replicate our main analysis using a sample that includes only post-reproductive individuals (allowing us to drop the two-stage modelling approach). Finally, we redo all analyses using conventional linear regression models. Results are discussed in the electronic supplementary material, S4.1, S4.2 and S4.3.

## 3. Results

### (a) Findings under various definitions of marriage success

#### (i) Bateman’s first principle

Sex-specific levels of variation in RS as measured with the opportunity for selection metric among the subset of individuals of age 45 and older (sample size: *n*_*m*_ = 171 men and *n*_*f*_ = 176 women) are: *I*_*m*_ = 0.30 (0.24, 0.36) and *I*_*f*_ = 0.21 (0.17, 0.25), for males and females, respectively. There is evidence of a sex difference in variance in RS, log(*I*_*m*_/*I*_*f*_) = 0.34 (0.07, 0.63). Note that values in parentheses throughout are 90% credible/confidence intervals (sometimes calculated using bootstrap resampling); if a 90% interval does not overlap zero, there is a smaller than 5% chance of the parameter having a value of opposite sign. Following the Fisherian expectation, average male, 6.2 (5.7, 6.6), and female, 6.0 (5.0, 6.3), RS is balanced.

#### (ii) Bateman’s second and third principles

To test Bateman’s second and third principles with various measures of marriage success, we examine each measure sequentially (table 1; and see the electronic supplementary material, S3.5 for full discussion). Considering only spouse number, we find no evidence of a difference in the opportunity for sexual selection between men and women of age 45 or older: log(*I*_*s*__*m*_/*I*_*s*__*f*_) = −0.16 (−0.43, 0.11) (table 2; *n*_*m*_ = 171, *n*_*f*_ = 176). Nor, in the corresponding regression model using only spouse number, is there evidence of a reliably positive relationship between spouse number and RS for either males, *β*_*m*_ = 0.05 (−0.06, 0.17), or females *β*_*f*_ = 0.04 (−0.05, 0.14) (table 1; *n*_*m*_ = 447, *n*_*f*_ = 627). Likewise, we fail to find any indication of sex differences in the effects of spouse number on RS, *β*_*m*_ − *β*_*f*_ = 0.01 (−0.14, 0.15) (table 2).

**Table 1.** Bateman analysis under various definitions of marriage success. The section labelled principle 2, MS provides the opportunity for sexual selection metric, *I_s_*, for the various included definitions of marriage success for each sex. The data used to calculate the opportunity for sexual selection metric come from individuals of age 45 or over (*n*_*m*_ = 171, *n*_*f*_ = 176). In the section labelled principle 3, Bateman gradient, we present the estimated regression coefficients: an intercept and the elasticities on age, spouse number (*N*), and—sometimes weighted—marital years, (*Y*), for males and females in each of the six statistical models. Each row presents the results of an independent model using a different measure, or combination of measures, of marriage success. A blank cell in the table indicates that the corresponding variable was not included in the model presented on that row. The section labelled WAIC contains the WAIC scores used for model comparison. Model comparisons are specific to sex. The term WAIC gives the WAIC information criteria, the term Δ gives the WAIC difference relative to the best model in the set, and *ω* gives the corresponding WAIC weight. The data used to calculate elasticities and WAIC scores come from the set of individuals with at least a single year of marriage. Intervals on the opportunity for sexual selection metrics are bootstrapped confidence intervals; otherwise, the values reflect posterior credible intervals. All intervals are 90% intervals.

		principle 2, MS	principle 3, Bateman gradient				WAIC		
		*I*_s_	intercept	age	spouse number (*N*)	marital years (*Y*)	WAIC	Δ	*ω*
spouse number	M	0.28 (0.23, 0.32)	−2.5 (−2.91, −2.09)	1.1 (0.99, 1.21)	0.05 (−0.06, 0.17)		1941.63	238.45	0
	F	0.32 (0.26, 0.39)	−2.21 (−2.47, −1.93)	1.03 (0.95, 1.1)	0.04 (−0.05, 0.14)		2471.11	158.06	0
marital years: unweighted	M	0.25 (0.2, 0.29)	−0.02 (−0.47, 0.45)	−0.33 (−0.53, −0.13)		0.88 (0.77, 1)	1776.96	73.78	0
	F	0.19 (0.15, 0.23)	−1.32 (−1.65, −1.03)	0.4 (0.24, 0.53)		0.46 (0.37, 0.55)	2391.22	78.17	0
marital years: marriage timing weights	M	0.18 (0.13, 0.22)	−0.55 (−1.65, 0.87)	−0.06 (−0.31, 0.17)		0.92 (0.8, 1.02)	1728.72	25.54	0
	F	0.09 (0.07, 0.13)	−0.61 (−1.82, 1.56)	0.4 (0.26, 0.54)		0.57 (0.45, 0.68)	2313.31	0.26	0.35
marital years: spousal quality weights	M	0.19 (0.15, 0.24)	−1.1 (−1.92, −0.15)	0.07 (−0.13, 0.24)		0.93 (0.81, 1.03)	1713.94	10.76	0
	F	0.14 (0.1, 0.17)	−0.84 (−1.73, 0.61)	0.39 (0.24, 0.52)		0.52 (0.43, 0.61)	2348.09	35.04	0
marital years: both weights	M	0.18 (0.14, 0.23)	−1.05 (−2.08, 0.67)	0.12 (−0.07, 0.33)		0.94 (0.84, 1.05)	1704.4	1.22	0.35
	F	0.09 (0.07, 0.13)	−0.65 (−1.75, 1.75)	0.41 (0.26, 0.55)		0.56 (0.45, 0.67)	2313.95	0.9	0.25
full model	M	0.18 (0.14, 0.23)	−0.87 (−1.97, 2.59)	0.08 (−0.13, 0.28)	−0.12 (−0.21, −0.02)	0.98 (0.87, 1.1)	1703.18	0	0.65
	F	0.1 (0.07, 0.13)	−0.72 (−1.79, 1.93)	0.4 (0.27, 0.54)	0.1 (0, 0.18)	0.57 (0.46, 0.68)	2313.05	0	0.4

**Table 2.** Male-to-female contrasts under various definitions of marriage success. The section labelled principle 2, MS provides the male-to-female contrast in inequality in marriage success. The section labelled principle 3, Bateman gradient provides the male-to-female contrast in the effects of spouse number and—sometimes weighted—marital years on RS. The symbol *I_s_* refers to the opportunity for sexual selection. For the opportunity for sexual selection metric, the contrast *δ*( · ) indicates the difference in the log of the male and female values—e.g. $\log(I_{s_{m}})-\log(I_{s_{\;f}})$; for the regression parameters on spouse number (*N*) and marital years (*Y*), it equals the difference of male and female values—e.g. $\beta_{N_{m}}-\beta_{N_{\;f}}$. Each row presents the results of an independent model using a different measure, or combination of measures, of marriage success. A blank cell in the table indicates that the corresponding variable was not included in the model presented on that row.

	principle 2, MS	principle 3, Bateman gradient	
	*δ*(*I*_s_)	*δ*(*N*)	*δ*(*Y*)
spouse number	−0.16 (−0.43, 0.11)	0.01 (−0.14, 0.15)	
marital years	0.26 (0, 0.53)		0.42 (0.27, 0.57)
marital years: marriage timing weights	0.63 (0.22, 1)		0.35 (0.2, 0.51)
marital years: spousal quality weights	0.33 (−0.01, 0.66)		0.4 (0.25, 0.54)
marital years: both weights	0.65 (0.27, 1.08)		0.38 (0.23, 0.53)
full model	0.64 (0.28, 1.08)	−0.22 (−0.35, −0.09)	0.4 (0.24, 0.57)

However, when marriage success is measured using marital years and weighting functions for both marriage timing and spousal age—the measure we call *effective marital years*—we find much more structured variation in reproductive and marriage success. The opportunity for sexual selection metric in this case suggests a reliable pattern of increased male relative to female variance in marriage success, log(*I*_*s*__*m*_/*I*_*s*__*f*_) = 0.65 (0.27, 1.08) (table 2; *n*_*m*_ = 171, *n*_*f*_ = 176). The relationship between marriage success and RS (for males, *β*_*m*_ = 0.94 (0.84, 1.05), and females, *β*_*f*_ = 0.56 (0.45, 0.67); table 1) is as is generally observed in non-human mammals, where the effect is reliably larger in males than females, *β*_*m*_ − *β*_*f*_ = 0.38 (0.23, 0.53) (table 2; *n*_*m*_ = 447, *n*_*f*_ = 627).

Considering the Watanabe–Akaike information criterion (WAIC) comparison, we note that inclusion of both weights improves predictive accuracy for both males and females relative to simpler models. Estimates of the sex-specific weighting functions are presented in the electronic supplementary material, S3.4. These functions and the ΔWAIC values presented in table 1 suggest that male fitness is tightly linked to the age of acquired spouses, and that female fitness is tightly linked to the timing of spousal acquisition.

### (b) Considering male and female pathways

To investigate if men and women can pursue unique pathways to maximize RS, we fit a model that includes both spouse number and effective marital years. The results of this full model are included in the bottom panels of tables 1 and 2.

**Figure 1.**
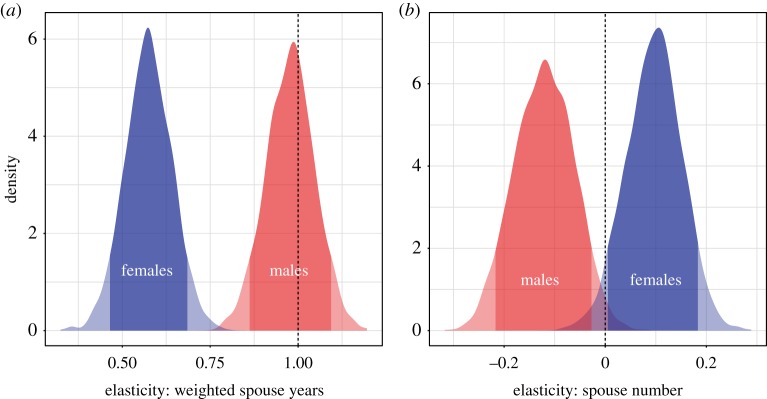
Elasticity estimates from the full model. The light shaded regions plot the posterior distributions, and the dark shaded regions plot the 90% posterior density intervals. We note that: (i) for males, the elasticity of RS with respect to effective marital years includes 1.0, the value of direct proportionality, while the corresponding estimate for females remains distant from this value (panel *a*); and, (ii) for males, the elasticity of RS with respect to spouse number is reliably negative, while the corresponding estimate for females is reliably positive (panel *b*). (Online version in colour.)

In both sexes, effective marital years have a reliably positive effect on RS, controlling for spouse number. And, as expected, the elasticity of RS with respect to effective marital years is reliably larger in men than in women (figure 1*a*). Regarding the effects of spouse number, however, we find diverging estimates for men and women. Women benefit from increasing spouse number, controlling for effective marital years; men, by contrast, pay a reproductive cost to increasing spouse number, controlling for effective marital years (figure 1*b*). There is a reliable sex difference in this effect (table 2). Note, however, that the magnitudes of the elasticities of RS with respect to effective marital years are larger than the elasticities of RS with respect to spouse number, indicating that variance in RS is better explained as a function of effective marital years.

## 4. Discussion

Our approach to the study of Bateman’s principles goes beyond simple contrasts of the benefits of multiple mating for males and females, and instead considers the paths through which multiple mating can affect RS. Focusing on a population showing no evidence of demographic transition (electronic supplementary material, S1.4), we find support for Bateman’s three principles, insofar as: (i) men show more variation in RS than do women, (ii) men show more variation in mating success (as marital years) than do women—although the effect only reaches high reliability upon accounting for marriage timing and spousal quality, and (iii) marital years are a stronger predictor of male than female RS. However, we also find that Pimbwe women achieve reliable fitness gains as a function of increasing spouse number (holding constant effective marital years), while Pimbwe men, by contrast, face reliable costs to their fitness from increasing spouse number (again holding constant effective marital years). Although cases where females benefit from multiple mating have been found across the animal kingdom (reviewed in [23,32]), until now the only indication of such patterning in humans has come from multiple paternity societies where children with multiple socially recognized fathers show higher survival [17,21]. We note the limitation, however, that even in this population, where successful extra-pair matings typically produce new, ethnographically observable marital partnerships, our data are based on (dyadically cross-checked) self-reports of reproduction and marriage that cannot ultimately substitute for genetic paternity data (see the electronic supplementary material, S1.2 for additional discussion).

### (a) Measuring mating success

There has been much debate over how to measure the strength of sexual selection. In empirical work across populations, proxies include mating rates [33], anisogamy [34], operational sex ratios [2,35], potential reproductive rates [36], time-in versus time-out [37] and (for humans) prescribed mating system [3]. Within-population analyses often rely on number of mates (variously defined, see Anthes *et al.* [8]) or, in the case of humans, number of marital partners (e.g. [9]). We have shown how in a species with potentially long-duration coresidential pairbonds structured around reproductive relationships, it can be more informative to use *marital years* as a proxy for marriage success [13], rather than relying simply on *spouse number*, as has been the standard practice in evolutionary demography; in fact, using spouse number alone, we would have failed to detect that the effect of mating success on RS is stronger in males than females. More generally, we draw attention to the use of unpacking the components of mating success. Although we explore this issue empirically in humans, the innovation has broader applicability, particularly insofar as the most appropriate metrics for studying sexual selection will vary across different systems [7]. Finally, we note that a broader formal understanding of sexual selection would benefit from investigating how the components of mating success influence reproduction within a model that includes mate choice and life-history dynamics [38].

Given that both sexes face potential trade-offs in the quality, quantity and duration of their partnerships (e.g. with respect to fecundity, investment and offspring quality [39]) a measure of mating success incorporating two dimensions (number of years partnered and number of unique partners) can help track sex-specific strategies. In so doing, we reveal hitherto under-appreciated aspects of female strategies in humans, with relevance to primates more broadly [17]. Our results are consistent with the idea that women may seek different kinds of mating relationships depending on the quality of assistance they can expect from spouses or others [40] and the amount of resources they can accrue [41]. Our results also suggest that men face trade-offs between investing time and resources in the acquisition of new spouses and in maintaining stable long-term relationships that produce thriving, high-status offspring [42,43]. Beyond humans, we contend that consideration of the distinct effects of mate number and partnership duration has implications for species with breeding systems where the delay to offspring recruitment is long relative to the time-span between partnerships—such systems include those where males frequently commit infanticide [18], and those where males and females can both benefit from switching mates mid season (e.g. shore birds [44])—in addition to those species which, like humans, have long-term partnerships and extended windows of parental care.

### (b) Accounting for the unusual patterning in the Pimbwe

As evolutionary reasoning penetrated the human sciences, researchers began to argue that human strategies in mating and marriage are a product of sexual selection consistent with the Darwinian–Bateman paradigm (e.g. [45]). Indeed, men generally show higher variance in fitness and number of mates (or spouses) than do women (reviewed in Brown *et al*. [3]), even in institutionally monogamous societies (e.g. [5]), and obtain greater fitness benefits from multiple mating. That said, there is debate over the significance of sexual selection for behavioural and psychological traits in our species (e.g. [46,47]). Given contemporary interest in the patterning of sexual selection [35,37,48–50], potentially unusual cases like the Pimbwe bear scrutiny.

As in a prior study [10], we find only weak evidence of sex differences in variance in RS when comparing post-reproductive men to post-reproductive women (the effect holds for individuals of age 45 or older, but vanishes for individuals over age 55—see the electronic supplementary material, S4.1). We speculate that this pattern results from the unreliable agricultural productivity in the region paired with restricted access to natural resources. These conditions lead to variable levels of resource access, both between men—increasing inequality in male provisioning over short time periods—and within men over time—damping the potential for strong and persistent inequalities in RS. Sex differences in variation in marriage success are most prominent when weighted marital years are analysed, indicating that variation in marriage success among Pimbwe men emerges most acutely from variation in the duration of time in which they have access to younger, more-fertile wives, rather than from variation in number of spouses or marriage duration *per se*. The stronger positive effect of marital years on RS in men relative to women reflects the ability of men to reproduce later in life than women and a tendency of men to take considerably younger women as second wives [10]. The negative effect of spouse number on RS holding constant marital years for men may be a result of prevalent female-initiated divorce and/or the costs of dividing a fixed set of resources across a wider resource sharing pool [29]. This being said, adding sequential or concurrent wives can be a fitness-enhancing strategy for men in Mpimbwe, but only so long as relationship duration is not compromised by pursuit of additional partners.

Our most parsimonious inference for the unusual patterning of spouse number on RS, with women benefiting more than men from multiple spouses (holding constant total marriage duration), is that many Pimbwe women switch partners to improve their economic circumstances. Possible mechanisms include women ‘trading up’ for better quality spouses (consistent with a meta-analysis of monogamous birds [51]), adaptive responses to high variability in territory quality (the ‘musical chair hypothesis’ [52]), responses to unpredictable environmental fluctuations [53], and/or the possibility that copulation rates increase after the formation of new relationships. Alternatively, this pattern might reflect reverse causality, whereby particularly fecund women attract more partners—a dynamic from which we cannot infer sexual selection on women [22,23]. In support of the ‘trading up’ idea, ethnographic observations and analyses of Pimbwe data indicate that material resources are critical to child survival [54], that prevalent conflicts over theft of shared household goods (such as crops in the field, grain in the store, or clothes and cash [27]) commonly trigger spousal conflict and divorce, and that there is great variability in economic productivity—both between men, and within men over time. Additionally, in-laws are highly valued as cooperative partners [27], suggesting a further benefit to having multiple spouses sequentially with affines who maintain interest in genetically related offspring even after a divorce. These explanations for the patterning of the results are speculative, but there are parallels in African ethnography: in Zambia women marry (and remarry) in search of supportive husbands [55], and in Malawi and Tanzania women use sexual relations to negotiate economic dependencies with multiple men [56,57]. Kaplan & Lancaster [42] make similar arguments for the instability of marriage under conditions of economic uncertainty.

### (c) Challenges and opportunities for studies of sexual selection

Our findings highlight specific challenges and opportunities with respect to the study of sexual selection in humans. First, we acknowledge that marriage is not the same as mating. Insofar as Pimbwe couples form households based on sexual relationships, marriage data are probably more reliable than self-reported ‘mating’ data. Additionally, because pregnancies typically lead to co-residence, paternity (claimed and acknowledged) is generally public knowledge. Although marriage and mating success are not identical measures, we argue that models decomposing the number, duration, timing and quality of such partnerships provide novel insights into the operation of sexual selection in humans, and are generalizable to non-humans more broadly (see also the electronic supplementary material, S1.3). Furthermore, because mating in most human societies is regulated to a greater or lesser extent through the institution of marriage and norms concerning ‘legitimacy’ (see the electronic supplementary material, S1.2), studies of humans provide the opportunity to investigate the linkages between socio-ecological circumstances (e.g. wealth differences), cultural norms (e.g. legislation forbidding polygyny) and the structure of mating systems (e.g. [58]). These are exciting frontiers for anthropologists.

Second, our findings caution us to be more circumspect regarding the generalizability of the inferences to be drawn from a small set of biological facts. Sex differences in mammalian reproduction, where females alone incur the costs of lactation and pregnancy, render coherent logic [7] and strong empirical support [1] to Bateman’s three principles. That said, divergences from the standard pattern are not surprising, given the multitude of factors affecting the operation of sexual selection in both sexes [49]. Unusual patterns often point to unusual trade-offs. The lengthy dependency of juveniles on adults in humans, exacerbated in this case by precarious economic conditions in Mpimbwe, accentuate trade-offs between reproductive and parental effort for both women and men, and probably account for the nimble mate switching that some Pimbwe individuals engage in. Mammalian constraints are not the whole story.

Third, human societies, with their diverse ecological, economic, social and institutional arrangements, differ greatly with respect to sex-specific choosiness, competitiveness, and parental tendencies [3,43], offering potential for comparative study of these dynamics across populations (e.g. [29]). Furthermore, insofar as the ‘polygynandry’ inherent in serial monogamy generally weakens Bateman gradients for males and strengthens them for females (as seen here), this lends credence to current critiques of the generality of the effects of sexual selection on a range of human phenotypes [46,59].

Finally, it bears emphasizing that among the Pimbwe, reproductive inequality among women emerges not from reproductive suppression (as it does in some cooperative breeding birds and mammals [32]) but, more likely, from direct competition among women for access to resources, including good mates and multiple caretakers [24,40]. Such competition is likely to characterize many female mammals owing to the high costs of gestation, and particularly women on account of the length of offspring dependency [60]. Accordingly, this particular pattern of sexual selection may be most apparent where securing the necessities of life is hard, and/or where males vary markedly in their quality and resource holdings. The extent and patterning of sexual selection in relation to varying ecological conditions is a re-emerging focus of study (e.g. [61,62]), and could be tackled systematically with the mating success metrics introduced here.

## Supplementary Material

Appendix

## Data accessibility

The data and code supporting this article will be maintained at: https://github.com/ctross/batemanpimbwe.
