# Supplementary material for: Unpacking mating success and testing Bateman’s principles in a human population
Source: Proc Biol Sci. 2019 Aug 14;286(1908):20191516. doi: 10.1098/rspb.2019.1516 (PMC6710586; doi:10.1098/rspb.2019.1516)
Supplement: Appendix [file rspb20191516supp1.pdf]

SOM: Unpacking Mating Success and Testing Bateman's Principles in a Human Population

Contents

1 Notes about the database **1**
1.1 The data used 1
1.2 Paternity ascription and naming customs 2
1.3 Marriage and mating 3
1.4 Fertility rates 3
1.5 Scatter plots and correlations of the data 4

2 Notes about the statistical analyses **5**
2.1 Elasticities 5
2.2 Main model 7
2.2.1 Main equations 7
2.2.2 Random effects 9
2.2.3 Priors 10
2.2.4 Software implementation 12

3 Supplementary results from the main model **12**
3.1 Model fit 12
3.2 Probability of remaining never-married 13
3.3 RS of never-married individuals 13
3.4 Timing and quality curves 14
3.5 Bateman's second and third principles (Full presentation of results) 15

4 Robustness checks **16**
4.1 Age threshold of 55 years for variance measures 16
4.1.1 Opportunity for selection measures 16
4.1.2 Opportunity for sexual selection measures 16
4.2 Simplified regression model, only adults of age 45 and over . . . 16
4.3 Simple linear regression models 17

1 Notes about the database

1.1 The data used

Our database is built from more than 20 years of longitudinal data collection. In this analysis, we use these longitudinal records to construct year-by-year

marriage histories for each individual. All individuals are censored at last household visit, and appear only once, as a single row, in the completed database. Therefore, our models do not require adjustments for repeated measurements.

In any given year, there were sometimes inconsistencies in the collected data—for example, conflicting records as to the year in which a wife and ex-husband claimed their relationship began—these conflicts were typically adjudicated at subsequent visits or with relatives or neighbors.

The database constitutes an almost complete census of a single village over the time-period of investigation. If households were missed (due to temporary travel absence, sickness, or a witchcraft case—the latter leading individuals to withdraw from social interactions) data were collected from neighbors and/or in subsequent visits. Village census (excluding members of the migrant Sukuma community who live outside of the main village cluster but on village land) varied from 1,001 (in 1995) to 1,540 (in 2010), with an almost constant sex ratio of about 98 males per 100 females.

RS is measured as the number of offspring reaching their fifth birthday, after which mortality is low. To measure the effects of marriage success on RS, we consider: i) number of spouses ever married (this ranges between 0-5 for both women and men); and, ii) the number of years a focal individual has been married. For men, who can be married to >1 spouse at a time in the case of concurrent polygyny, this value can exceed the number of years for which they have wives. We draw on recent methodological advances using Gaussian random fields [1] to explicitly account for the effects of: iii) the timing of marriages, and iv) the age of each spouse in each year.

The dataset and code supporting this article are included the Supplementary Materials and will be maintained at https://github.com/ctross/batemanpimbwe.

1.2 Paternity ascription and naming customs

It is possible that there might be a fraction of misattributed paternity due to 'hidden copulations' in this population, and how these cases are coded could differentially influence the apparent variance of male and female fitness. Genetic measures of paternity are not available for this population, but repeated household censuses over 20 years reveal remarkable stability to the claimed paternity of children; children are seen as wealth in much of Africa ("wealth in people" [2]) and paternity is rarely denied by men who have sired offspring. Although women might infrequently manipulate attributions of paternity, in such cases there is normally much public discussion on the topic; typically a consensus is reached before the child reaches 3 years of age, often on the basis of physical resemblance. Pimbwe name their children at birth with a "traditional"

name, typically the traditional name of a recently deceased ancestor or a nickname depicting some characteristic of the child (e.g., "Tabu," Troublesome). Very soon after birth, the child is also given the name of his/her father; if the mother fails to divulge the name of the child's father, the child takes the name of his mother's father. As the child grows she or he is usually given a religious name (for schooling or other administrative purposes). While religious names can change over the years (in approximately 3% of cases, e.g., Maria to Marietta, Marisella or Meri, or to an entirely new name by choice), Pimbwe names generally remain stable in our demographic records. A child's father's name changed only in 11 of 1,587 cases. In each of these cases, an initially recorded maternal grandfather's name was replaced by the name of the child's presumed father, because the man in question claimed the child, typically marrying the child's mother. In two cases there was no resolution on a father's name, and the child stayed with his maternal grandfather's name.

1.3 Marriage and mating

Mating in humans is often regulated through the concept of legitimacy conferred through marriage. We view this as an opportunity for, rather than a challenge to, theorizing sexual selection. Humans are unique in institutionalizing formal norms that constrain mating access. These constraints are variable, both between populations [3], and even within populations, where there may be different kinds of "marriages" [4, 5]. Furthermore, marriage can both constrain extra-pair mating and result from extra-pair mating. Accordingly, evolutionary perspectives on sexual selection in humans prefer not to treat marital institutions as entirely exogenous, but rather focus on the norms that pertain to reproduction as coevolving with strategies dictated by individual fitness interests (e.g., [6, 7]). In short, while marriage and mating are not equivalent, marriage is a particularly human contract that can shed light on the operation of sexual selection in our species.

1.4 Fertility rates

Initial analysis of the data suggest that there is no evidence of demographic transition in this highly rural population. There is no evidence of a decline in the rate at which women or men (at or over age 30) produce surviving children as a function of birth year (See Table S1)—in fact, the estimated coefficient of year of birth for women is significantly positive. The R^2 coefficient is very small in both males and females, which itself indicates that birth year plays little role in explaining fertility rate. See also the scatter plots in Figure S1.

While simple analyses such as this can be biased by differential survivorship of individuals contingent on their fertility, and differential reporting of births contingent on respondents' age, the lack of any evidence of a negative association between birth year and fertility suggests that demographic transition in Mpimbwe is minimal.

Moreover, and quite contrary to demographic transition, we find that the frequency of polygyny is actually increasing in recent years, suggesting that males are still aiming to increase reproductive output by acquiring multiple wives. See Figure S2.

Accordingly, we refer to the study population as being largely characterized by natural fertility.

[Table S1 about here.]

[Figure S1 about here.]

[Figure S2 about here.]

1.5 Scatter plots and correlations of the data

To gain a sense of the data distributions, we plot bivariate scatters and correlation coefficients for all individual-level data used in the analysis. Figures S3-S6 present plots for various subsets of data: 1) those ever married, 2) those at least 45 years of age, 3) those who have married more than once, and 4) the full dataset.

[Figure S3 about here.]

[Figure S4 about here.]

[Figure S5 about here.]

[Figure S6 about here.]

Note, however, that high-dimensional relationships can be difficult to see in raw scatters. Most variables included here are influenced by age. Given this consideration, we suggest that Fig. S4, limited to individuals of 45 years and over, is most informative.

2 Notes about the statistical analyses

2.1 Elasticities

In the main text, we use elasticity parameters, rather than standard slope coefficients, to estimate the relationship between an input variable (marriage success) and an output variable (reproductive success). This is required to ensure a properly specified statistical model that reflects the constraints on the plausible values of the data.

An elasticity parameter, ω, for example, represents the percent change in one variable, y, as a function of the percent change in another, x. It provides an estimation of the relationship between variables that allows for diminishing marginal returns. It is defined as:

$$\omega = \frac{\partial \log y}{\partial \log x} = \frac{\partial y}{\partial x}\frac{x}{y} \tag{1}$$

A slope parameter, β, by contrast, assumes that there are never diminishing marginal returns between variables:

$$\beta = \frac{\partial y}{\partial x} \tag{2}$$

Notice, that in a standard linear regression model like:

$$RS \sim \text{Normal}(a + b \cdot \text{Age} + c \cdot \text{Spouse Number} + d \cdot \text{Marital Years}, \sigma) \tag{3}$$

the right-hand side implies that RS can be real-valued or even negative. Also, the coefficients controlling the effects of age, spouse number, and marriage years are constant for all values of the inputs. These are assumptions that we know are discordant with the data generating process *a priori*. There is both theoretical and empirical [8] evidence that there are typically diminishing marginal returns to each of these input variables in humans—for example, we don't expect the effect of a marginal decade of age on RS to be the same for a 20 year old woman as for an 80 year old woman. Therefore, we do not use a model which assumes this to be the case, because doing so can easily lead to incorrect estimates of the values we hope to measure. The main model we use—essentially a Cobb-Douglas production function—is standard in economics for modeling production [9] and reproduction [10] where outcomes are non-negative and diminishing marginal returns to inputs are possible.

While theoretical models of reproduction often consider the linear relationship between variables, such approximations are normally justified in light of simplifying assumptions that don't hold in empirical cases. For data on human

reproduction, where diminishing marginal returns are commonly found, accurate estimation of the relationship between variables suggests the use of elasticities, so that the slope $\frac{\partial y}{\partial x}$ depends upon the magnitude of the independent variable being evaluated. We note here that when we consider the AIC of the basic linear model:

$$RS \sim \text{Normal}(a + b \cdot \text{Marital Years}, \sigma) \tag{4}$$

and the AIC of a simple model that instead uses elasticities:

$$RS \sim \text{Poisson}(\exp(a + b \cdot \log(\text{Marital Years}))) \tag{5}$$

for males, the linear model has an AIC of 218.91 points more than the model which uses elasticities. For females, the difference is starker at 251.86. In other words, by using the assumption of a constant slope, we severely reduce our predictive accuracy relative to a better-specified generative model.

Our use of elasticities, however, does not mean that our methods cannot speak to the simple slope of RS on marriage or mating success. Should a slope estimate at a given point be desired, it is recoverable by taking the partial derivative of the reproduction function with respect to a given input. This involves nothing more than a transformation of our parameter estimates.

For example, using a simple function for reproduction: $r \sim \alpha x^{\gamma} y^{\omega}$, where r is reproductive output, $x > 0$ is age, $y > 0$ is years married, $\alpha > 0$ is an intercept and ω and γ are elasticities, the slope of years married on reproduction, for individuals of a given age class, is:

$$\beta = \alpha \omega y^{\omega - 1} x^{\gamma} \tag{6}$$

As an example of this transformation, we present the corresponding male and female values of the implied slope coefficients from our model of marital years described in the main paper (Figure S7). We also present the corresponding plot from a robustness check using only individuals of age 45 or older, dropping the two stage model (Figure S8, and see discussion in section 4.2)—finding essentially identical inferences using either methodological approach. Here we find that because of diminishing marginal returns to years married for females, and approximately constant marginal returns to years married for males, the slope of reproductive success on marriage success starts out approximately equal for males and females of a given age, but a sex difference becomes pronounced as number of years married increases.

[Figure S7 about here.]

[Figure S8 about here.]

2.2 Main model

To accommodate the fact that some of our empirical data show strong signs of non-linearity (specifically in the form of diminishing marginal fitness returns to marriage success for females), we measure the Bateman gradient using an *elasticity* parameter, which indicates the percent change in RS with respect to the percent change in marriage success. This is a standard approach in economics to modeling reproduction and marriage [8], where diminishing marginal fitness returns to marriage success occur and would lead to inaccurate estimates of standard slope coefficients. Standard slope coefficients, however, can be calculated from our parameter estimates assuming a fixed level of inputs (as demonstrated above in section 2.1). We note that our reproductive success data also show strong signs of zero-inflation and over-dispersion (Figure S9). This leads standard linear regression models to fail quality checks—as we show at the end of section 4.3.

2.2.1 Main equations

To appropriately model our zero-inflated reproductive success data (see Figure S9), we use a two-stage modeling framework [1] and a modeling structure that lets us decompose the paths through which spouse number affects RS (see Figure S10).

[Figure S9 about here.]

[Figure S10 about here.]

First, we model a binary indicator representing if individual, i, has achieved at least a single year of marriage, $M_{[i]}$, as a function of age:

$$M_{[i]} \sim \text{Bernoulli}(\text{logistic}(\alpha_{[1,S(i)]} + \alpha_{[2,S(i)]} \log(E_{[i]}))) \tag{7}$$

where $E_{[i]}$ is the *exposure time* to the possibility of reproduction—i.e., years lived in the interval between age 11 and death/censoring—and $S(i)$ is a function returning the sex of individual i. See the results of this submodel in section 3.2.

In cases where $M_{[i]} = 0$, we expect (and find, see section 3.3) that the reproductive success, R, of individual, i, will generally be equal to zero as well, given the nature of sexual reproduction. Cases of $M_{[i]} = 0$, and the corresponding cases of $R_{[i]} = 0$, are tightly linked to age, with the probability that $M_{[i]} = 0$ itself approaching zero for individuals over age 30 of either sex (see section 3.2). Additionally, a very small number individuals with a documented

$M_{[i]} = 0$ do have $R_{[i]} > 0$ (see section 3.3). In such cases, the observation of $M_{[i]} = 0$ reflects the fact that the focal individual reproduced without marrying. We model the distribution of $R_{[i]}$, conditional on $M_{[i]} = 0$ using a separate model from cases where $M_{[i]} = 1$, since cases of the former are heavily influenced by very young individuals.

In cases where $M_{[i]} = 1$, we fit our main model linking marriage success and reproductive success. Specifically, we model the reproductive success, R, of individual, i, using a negative binomial outcome distribution [8]:

$$R_{[i]} \sim \text{Negative Binomial}(\mu_{[i]} B_{[S(i)]}, B_{[S(i)]}) \tag{8}$$

where the term $\mu_{[i]} B_{[S(i)]}$ defines the shape parameter of a Gamma distribution, and $B_{[S(i)]}$ defines the inverse scale parameter. This is equivalent to using a Gamma-Poisson mixture model, which has been recommended for modeling *over-dispersed* fertility-related outcomes—i.e., where the variance exceeds the mean—which are commonly found in polygynyous societies [11]. We can then define a model of the mean of RS, $\mu_{[i]}$, using a standard log link function:

$$\log(\mu_{[i]}) = \beta_{[1,S(i)]} + \beta_{[2,S(i)]} \log(E_{[i]}) + \beta_{[3,S(i)]} \log(N_{[i]}) + \exp(\beta_{[4,S(i)]}) \log(Y_{[i]}) \tag{9}$$

where the new variables in the regression are: spouse number, $N_{[i]}$, and effective marital years, $Y_{[i]}$—this variable is constructed as described below. Note that the regression parameters, β, are unique to the sex of individual i, and that the elasticity on $Y_{[i]}$ is constrained to be positive, to ensure identification given the reflection invariance that could otherwise arise from the multiplication of parameters.

The variable $Y_{[i]}$ (number of "effective marital years") is obtained for an individual by calculating a weighted sum of the number of years in which he or she has been married to each of his or her spouses. More formally, $Y_{[i]}$ is the sum:

$$Y_{[i]} = \sum_{e=1}^{E_{[i]}} \sum_{n=0}^{N_{[i,e]}} \begin{cases} 0, & \text{if } n = 0 \\ \underbrace{\text{logistic}(\theta_{[S(i)]} + \nu_{[e,S(i)]})}_{\substack{\text{Value to a focal individual of a} \\ \text{marital year } at \text{ a given age}}} \cdot \underbrace{\text{logistic}(\phi_{[S(i)]} + \psi_{[A(n,i,e),S(i)]})}_{\substack{\text{Value to a focal individual of marriage} \\ \text{to spouse } of \text{ a given age}}}, & \text{if } n > 0 \end{cases} \tag{10}$$

where the first factor gives the estimated value to the focal individual of having a spouse *at* a given age (timing weight) and the second factor gives the estimated value to the focal individual of having a spouse *of* a given age (spousal quality weight). We note that effective marital years and spouse number in the main model are not strongly correlated (for men $\rho = 0.3$; for women $\rho = 0.05$).

In the first factor, θ is an intercept and ν is an age-specific random effect. In the second factor, ϕ is an intercept and ψ is an age-specific random effect. The function $A(n, i, e)$ gives the age index of the n-th spouse of individual i, in individual i's e-th year of life. Eq. 10 imposes the following: 1) if an individual is not married in a given year, the number of quality-weighted spouse years is not incremented in that year; 2) the maximum possible value of a quality-weighted spouse year is 1, and is approached when an individual of a given sex is married during their peak reproductive period to a spouse also at their peak reproductive period; and 3) if an individual is married, the number of quality-weighted spouse years is incremented, but the weights on spousal quality and marriage timing may reduce this value to something less than 1, but greater than 0.

To test our predictions concerning how one's definition of mating success affects the apparent Bateman gradient, we can refit the model, varying our definition of $Y_{[i]}$. For example, we can replace $\text{logistic}(\theta_{[S(i)]} + \nu_{[e,S(i)]})$ and/or $\text{logistic}(\phi_{[S(i)]} + \psi_{[A(n,i,e),S(i)]})$ with the constant value 1, and investigate how the relationship between reproductive success, R, and marriage success, Y, changes as we account for the timing of marriages and the quality of spouses, alone and in combination. All random effects in this model are estimated using Gaussian random fields (see details in section 2.2.2).

In cases where $M_{[i]} = 0$, we fit a separate model linking marriage success and age (also referred to as exposure time). Because marriage does not exactly correspond to mating, we have to make a special allowance that never-married individuals can reproduce. So, as in the main model, we model the reproductive success, R, of individual, i, again using a negative binomial outcome distribution [8].

$$R_{[i]} \sim \text{Negative Binomial}(\mu_{[i]} B_{[S(i)]}, B_{[S(i)]}) \tag{11}$$

However, we now define an independent, simplified model of mean RS in this class of individuals:

$$\log(\mu_{[i]}) = \hat{\beta}_{[1,S(i)]} + \hat{\beta}_{[2,S(i)]} \log(E_{[i]}) \tag{12}$$

where the only predictor is $E_{[i]}$, the age of individual i minus 11 years—i.e., the number of years in which individual i has been old enough to theoretically reproduce. See the results of this submodel in section 3.3.

2.2.2 Random effects

We model the random effects vectors ν and ψ using Gaussian random field submodels, which allow for the effects marriage timing and spousal quality

to take on arbitrary functional forms, while still partially sharing information across neighboring parameters. More specifically, we model:

$$\nu \sim \text{Multi. Normal Cholesky}((0,\ldots,0)', \eta_\nu L_\nu) \tag{13}$$

$$\psi \sim \text{Multi. Normal Cholesky}((0,\ldots,0)', \eta_\psi L_\psi) \tag{14}$$

where $\eta_\nu, \eta_\psi \in (0,\infty)$ serve to scale variance, and L_ν and L_ψ are factors given by the Cholesky decomposition of the correlation matrices ρ_ν and ρ_ψ. The correlation matrices are defined using a distance decay function:

$$\rho_{\nu[i,j]} = \kappa_\nu \exp\left(-\tau_\nu \frac{(i-j)^2}{C^2}\right) \tag{15}$$

$$\rho_{\psi[i,j]} = \kappa_\psi \exp\left(-\tau_\psi \frac{(i-j)^2}{C^2}\right) \tag{16}$$

where $\kappa_\nu, \kappa_\psi \in (0,1)$ control the maximum correlations, $\tau_\nu, \tau_\psi \in (0,\infty)$ control the decay rates, and C is a constant which normalizes the maximum distance between age categories to 1.

The use of a multivariate normal distribution to generate correlated random effects carries several benefits: 1) it allows for partially-pooled estimation of marriage timing and spousal quality weights, 2) it imposes no *a priori* functional form (e.g., linear, quadratic, etc.) on these random effects, and 3) it allows for a reduction in the effective parameter complexity of the model by reducing the extent to which neighboring random effects parameters can vary independently.

2.2.3 Priors

We define weak priors over the top-level parameters. These priors reflect the fact that we have little information about the values of model parameters *a priori*, and allow the data and likelihood to dominate the posterior. Using subscripts of m and f for the male and female parameters, in the main regression equations, we use:

$$\alpha_m \sim \text{Normal}(0,5) \tag{17}$$

$$\alpha_f \sim \text{Normal}(0,5) \tag{18}$$

$$\beta_m \sim \text{Normal}(0,5) \tag{19}$$

$$\beta_f \sim \text{Normal}(0, 5) \tag{20}$$

$$\hat{\beta}_m \sim \text{Normal}(0, 5) \tag{21}$$

$$\hat{\beta}_f \sim \text{Normal}(0, 5) \tag{22}$$

$$B_m = 1/\hat{B}_m \tag{23}$$

$$B_f = 1/\hat{B}_f \tag{24}$$

$$\hat{B}_m \sim \text{Normal}(0, 10)T[0, \infty] \tag{25}$$

$$\hat{B}_f \sim \text{Normal}(0, 10)T[0, \infty] \tag{26}$$

The parameters on the random effects have weakly informative priors:

$$\theta_m \sim \text{Normal}(0, 5) \tag{27}$$

$$\theta_f \sim \text{Normal}(0, 5) \tag{28}$$

$$\phi_m \sim \text{Normal}(0, 5) \tag{29}$$

$$\phi_f \sim \text{Normal}(0, 5) \tag{30}$$

The correlation parameters have Beta priors:

$$\kappa_{\nu_m} \sim \text{Beta}(10, 2) \tag{31}$$

$$\kappa_{\nu_f} \sim \text{Beta}(10, 2) \tag{32}$$

$$\kappa_{\psi_m} \sim \text{Beta}(10, 2) \tag{33}$$

$$\kappa_{\psi_f} \sim \text{Beta}(10, 2) \tag{34}$$

The decay parameters have half-normal priors:

$$\tau_{\nu_m} \sim \text{Normal}(0,5)T[0,\infty] \tag{35}$$

$$\tau_{\nu_f} \sim \text{Normal}(0,5)T[0,\infty] \tag{36}$$

$$\tau_{\psi_m} \sim \text{Normal}(0,5)T[0,\infty] \tag{37}$$

$$\tau_{\psi_f} \sim \text{Normal}(0,5)T[0,\infty] \tag{38}$$

The variance parameters have half-normal priors:

$$\eta_{\nu_m} \sim \text{Normal}(0,5)T[0,\infty] \tag{39}$$

$$\eta_{\nu_f} \sim \text{Normal}(0,5)T[0,\infty] \tag{40}$$

$$\eta_{\psi_m} \sim \text{Normal}(0,5)T[0,\infty] \tag{41}$$

$$\eta_{\psi_f} \sim \text{Normal}(0,5)T[0,\infty] \tag{42}$$

2.2.4 Software implementation

Models are fit using Hamiltonian Monte Carlo [12] and the Stan 2.16.0 C++ library [13]. Markov Chain Conte Carlo methods are needed to accurately sample to posterior distribution of this complex, multi-level model. Models are implemented using an R [14] workflow, through the rstan interface. All code used in modeling is included in the Supplementary Materials folder. The code will also be maintained on `https://github.com/ctross/batemanpimbwe`.

3 Supplementary results from the main model

3.1 Model fit

Figure S11 gives the traceplots for each of the models fit in the main paper (ordered as in Table 1 of the main text). We see that in each model both chains appear to have converged to the same posterior region and to have mixed thoroughly.

[Figure S11 about here.]

3.2 Probability of remaining never-married

The first submodel of the main analysis investigates the sex-specific probability of remaining never-married as a function of age. We find that males have higher probability of remaining never-married until later in life than females. However, both males and females reach high levels of marriage probability by their mid-thirties (Figure S12).

[Figure S12 about here.]

3.3 RS of never-married individuals

From theory, we would expect reproductive success to follow a zero-inflated negative binomial generative model, where, for example, RS=0 in all cases where an individual has never acquired even a single mate, and RS $\in \mathbb{N}_0$ if an individual has ever acquired a mate. Because marriage is not equivalent to mating, however, we test this expectation, using a model that allows for never-married individuals to reproduce. Specifically, we model sex- and age-specific reproductive success among never-married individuals using a standard negative binomial model. We find that never-married females have reliably higher average levels of reproductive success than never-married males (Figure S13). For both males and females, never-married individuals—especially the young—are predicted to have near zero RS. The predicted mean number of offspring does not reach the value of one for females until age thirty, and for males it does not reach the value of one until after age 55 (Figure S13)—essentially confirming our above-stated expectation, and supporting our use of a zero-inflated negative binomial generative model. Figure S14 provides direct density estimates of RS among never-married individuals, and leads to exactly the same inference—although marriage is not equivalent to mating, our RS data differ by only a small margin from those that would be produced by a zero-inflated negative binomial model; nearly all density for RS in never-married males and females is concentrated on the value RS=0.

[Figure S13 about here.]

[Figure S14 about here.]

We note that estimates of male reproductive success, and particularly the reproductive success of never-married males, may be underestimated if such

males have sired offspring outside of the village where this study was conducted. Children born in non-marital relationships (either prior to marriage or with an extra-pair individual) are more likely to appear in a mother's than a father's record, for two reasons: first, a young child has a higher likelihood of living with its mother than its father, and therefore being observed as a household resident; second, on account of the above bias and the fact that some non-marital relations occur with individuals outside the focal village, illegitimate children born to the men of the focal village with outside women are more likely to be lost from the record. While such issues are unavoidable properties of studies of human fertility, the difference in average reproductive success for reproductively complete (age 45 or older) females and males in our sample is only -0.18, which is small in proportion to the average number of kids, 6.09.

Our main model—a zero-inflated negative binomial model—serves mostly to automatically adjust the sample to exclude individuals who are too young to reproduce. As a robustness check, we also analyze the relationship between RS and marriage success using only those individuals of age 45 or over. This eliminates the zero-inflation problem, and negates the need to use a two-stage model, or to restrict inclusion into the sample to only those individuals with at least a single spouse or marital year. The results of this model are generally comparable to those of the main analysis. See section 4.2 for details.

3.4 Timing and quality curves

In order to interpret our measures of marriage success in the main text, we present the estimated sex-specific weights for the value of having a spouse *at* a given age (marriage timing) and the value of having a spouse *of* a given age (spousal quality). With regards to the marriage timing weights, we note two key empirical results: 1) marriages occurring when individuals of either sex are younger ($<$ 40 years old) have larger implications for reproductive success than marriages which occur at older ages; and, 2) the reproductive value of marriages for males diminishes less with age than for females, as in indicated by the male curve (Figure S15.a) bottoming after age 60, while the same curve for females (Figure S15.b) bottoms earlier. These curves effectively show sex differences in age-specific reproduction for this population, reflecting, for example, the effects of menopause on the reproduction of females. These curves were estimated endogenously in the model, not hard-coded *a priori*; the fact that they reflect known patterns in humans give us confidence in the appropriateness of our modeling technique.

[Figure S15 about here.]

We note from the spousal quality curves that: 1) for males, acquisition of spouses who are younger (< 40 years old) has a larger impact on reproductive success than acquisition of older spouses, as indicated by the high density near value equals 1 for younger spouses, and high density near value equals 0 for older spouses (Figure S16.a); and, 2) for females also, acquisition of younger spouses (< 60 years old) has a larger impact on reproductive success than acquisition of older spouses (Figure S16.b).

[Figure S16 about here.]

The similar patterning of the estimated curves between Figures S15 and S16 are notable—and expected—given that the spousal quality curve for a male, for example, should theoretically follow a similar form as the marriage timing curve for a female.

3.5 Bateman's second and third principles (Full presentation of results)

In the main text, we commented on spouse number and the fully weighted model. Considering only spouse number, we found no evidence of a difference in the opportunity for sexual selection between men and women of age 45 or older. Nor, in the corresponding regression model using only spouse number in the full sample of individuals, was there evidence of a reliably positive relationship between spouse number and RS for either males or females. Likewise, we failed to find any indication of sex differences in the effects of spouse number on RS.

Defining marriage success using marital years rather than spouse number, however, we find marginally reliable evidence of a difference in variance in marriage success between males and females of age 45 or older: $\log(I_{sm}/I_{sf}) = 0.26\ (0.00, 0.53)$ (Table 2 in main text; $n_m = 171$, $n_f = 176$). Also, we now find reliably positive effects of marriage success on RS, for males, $\beta_m = 0.88\ (0.77, 1.00)$, and females, $\beta_f = 0.46\ (0.37, 0.55)$ (Table 1 in main text); $n_m = 447$, $n_f = 627$), and evidence that the effect is stronger in males than females, $\beta_m - \beta_f = 0.42\ (0.27, 0.57)$ (Table 2 in main text), as is predicted under the Bateman model.

Further inclusion of weighting functions on martial years leads to better predictive accuracy, as measured by WAIC, and yields stronger elasticity estimates, as we describe in the main text.

4 Robustness checks

4.1 Age threshold of 55 years for variance measures

Taking an age cut-off at age 45 might be considered too early as an indicator of overall fitness, especially for men. Accordingly, we calculate the opportunity for selection, and the opportunity for sexual selection, for a smaller sample of individuals older than 55 years of age.

4.1.1 Opportunity for selection measures

Sex-specific levels of variation in RS for men ($n_m = 97$) and women ($n_f = 104$) older than age 55 as measured with the opportunity for selection metric are: $I_m = 0.19$ $(0.14, 0.25)$ and $I_f = 0.20$ $(0.15, 0.25)$. There is no evidence of a sex difference in variance in RS, $\log(I_m/I_f) = -0.01(-0.41, 0.38)$. Considering only those men who are later into their post-reproductive period eliminates the sex-difference that was apparent among individuals older than 45. We suspect that this reflects a more recent increase in the frequency of concurrent polygyny (see, for example, Figure S2).

4.1.2 Opportunity for sexual selection measures

Here, using the same older sample, we examine the opportunity for sexual selection, specifically for spousal numbers and (weighted) marital years. Like the younger sample ($\geq$45 years), the older sample shows no sex difference in variation in spouse number. However, unlike in the $\geq$ age 45 sample (where we see a marginally reliable effect), the older sample also shows no sex difference in variation in marital years. But, as we show in Table S2, we see that as long as we include weights on the number of marital years, we detect increased male-to-female variance in mating success even in the subset of individuals over the age of 55—$n_m = 97$ and $n_f = 104$—replicating the result from the main analysis.

[Table S2 about here.]

4.2 Simplified regression model, only adults of age 45 and over

In the main text, we attempt to fully mine the information content of the database by using the full sample of individuals (those over age 11). This gives us a sample size of 447 males and 627 females, but introduces some added complexity in the modeling, by requiring a two-stage model design to deal with

zero-inflation [1]. Here, we present the results of a comparable analysis using only adults of age 45 and over. In this case, our sample size is reduced to 171 males and 176 females, but the issue of zero inflation is eliminated. In this case, we can simplify the statistics, omit the two-stage model, and avoid using the measure 'ever married' to permit inclusion into the main regression analysis. In this case, there are only four males and a single female with zero years married—possibly due to missing data or simply concealment of specific relationships by respondents—we therefore treat the marriage success measures of these individuals as if they were missing data, constrained to fall within the observed population-level distribution.

[Table S3 about here.]

[Table S4 about here.]

The results are presented in Tables S3 and S4, which correspond directly to Tables 1 and 2 of the main text. The qualitative results of the main text are largely unchanged. We do note that in this subset of data, the negative effect of spouse number on RS in the full model remains for males, but the positive effect for females disappears. The magnitude of the sex difference, however, remains mostly constant and this contrast remains reliable. The fact that the $\geq$45 sample shows a slightly different patterning than the full sample reported in the main paper suggests the possibility that the advantage of multiple spouses to Pimbwe women has become more prominent among younger and more contemporary women than it was in the past.

4.3 Simple linear regression models

We also replicate the main analysis using simpler tools. First, we use the same subset of individuals—those with at least one year of marriage—as we used in the main paper. The results are presented in Table S5. As we found in our main analysis, in the univariate models spouse number is not a reliable predictor of RS in men or women. Moreover, in the univariate models, marital years are a reliable predictor of RS in men and women—and the effect is roughly twice as large among men, just as we found in the main analysis using elasticities. Assuming that we use the methods described in section 2.1 to calculate slopes from elasticities—and evaluate these calculations for individuals of age 55 with 40 years of marriage—then the slopes derived from the elasticites estimated in the main text are: 0.163 (0.135, 0.190) for males, and 0.075 (0.059, 0.095) for females. These values are in near numerical correspondence with the

slopes estimated directly in the linear models. Finally, in the model with both predictors, we find that the effect of spouse number on RS holding constant marital years is smaller in males than in females, just as we found in the main analysis. There is a difference on this point between the OLS and Bayesian models in that the negative effect of spouse number on male RS in model (3) does not reach significance in the OLS model. The difference between this OLS model and the Bayesian model presented in the main paper is that the Bayesian model includes timing and spousal quality weights on the number of marital years, increasing the predictive accuracy of the model. The OLS model by contrast uses raw marital years.

[Table S5 about here.]

Next, we use the same subset of individuals as in section 4.2—those individuals of age 45 or over. The results of these models are presented in Table S6. As we found in our Bayesian analyses, in the univariate models spouse number is not a reliable predictor of RS in men or women. Moreover, in the univariate models, marital years are a reliable predictor of RS in men and women—and the effect is a little more than twice as large among men, just as we found in the Bayesian analysis using elasticities. Finally, in the model with both predictors, we find that males suffer from increasing spouse number, holding constant years married, while females benefit. There is a difference on this point between the OLS and Bayesian models in that the negative effect of spouse number on male RS in model (3) does not reach significance in the OLS model. As noted before, the Bayesian model includes timing and spousal quality weights on the number of marital years, increasing the predictive accuracy of the model. The OLS model by contrast uses raw marital years.

[Table S6 about here.]

Finally, we fit the OLS models using the whole dataset, including children as young as 11 years old with $RS = 0$ and martial years $= 0$. As we found in our Bayesian analyses, in the univariate models, marital years are a reliable positive predictor of RS in men and women—and the effect is a little more than twice as large among men. In the univariate models here, however, spouse number is now a reliable positive predictor of RS in both women *and* men. The effect is even slightly larger in males than females. As we expand on later, this result is likely driven by the model failing to correctly deal with zero-inflation. In the model with both predictors, we find that males still benefit more from increasing marital years, holding constant spouse number, than do females.

Females continue to benefit more than males from increasing spouse number holding constant years married, but, in this case, both effects are on the positive side of zero. Again, the Bayesian model includes timing and spousal quality weights on the number of marital years, increasing the predictive accuracy of the model. The OLS model by contrast uses raw marital years.

This last model would lead us to somewhat different conclusions than all other models presented in the main text and this supplement. In this set of models, for both sexes, the strong effects of spouse number on RS primarily reflect the marginal change in RS between those—frequently very young—individuals with zero spouses and those with at least single spouse, an effect seen in many other populations, e.g., [15, 16, 17]. In the model presented in the main paper, however, the effect of spouse number on RS primarily reflects the marginal change in RS between those individuals with a single spouse and those with more than a single spouse.

Note, however, that this last OLS model deals poorly with the zero-inflated outcome data, and the QQ-plots [18] of the residuals suggest that the assumptions of the linear regression analysis are violated—making the results of the model hard to evaluate. In Figure S17, note how the QQ-plots of the residuals for both male and female outcomes show a dramatic divergence of the distribution of model residuals from that which is assumed by the regression model. By contrast, if we use only those individuals age 45 and older, the QQ-plots in Figure S18 demonstrate that the residuals are distributed as assumed by the linear regression model. In unison, these figures suggest that our statistical inferences with OLS are valid in the case where only post-reproductive individuals are included in the analysis, but not in the case where standard OLS tools are applied to the full dataset. We suspect that some of the OLS results differ due to deviations from the assumption of normality.

[Table S7 about here.]

[Figure S17 about here.]

[Figure S18 about here.]

References

[1] McElreath, R. *Statistical Rethinking: A Bayesian Course with Examples in R and Stan*, vol. 122 (CRC Press, 2016).

[2] Guyer, J. I. Wealth in people and self-realization in Equatorial Africa. *Man* 243–265 (1993).

[3] Leach, E. R. Polyandry, inheritance and the definition of marriage. *Man* **55**, 182–186 (1955).

[4] Smith, M. G. Secondary marriage in northern Nigeria. *Africa* **23**, 298–323 (1953).

[5] Scelza, B. A. Female choice and extra-pair paternity in a traditional human population. *Biology Letters* rsbl20110478 (2011).

[6] Blurton Jones, N. *Demography and evolutionary ecology of Hadza hunter-gatherers* (Cambridge University Press, 2016).

[7] Hawkes, K., Rogers, A. R. & Charnov, E. L. The male's dilemma: increased offspring production is more paternity to steal. *Evolutionary Ecology* **9**, 662–677 (1995).

[8] Ross, C. T. *et al.* Greater wealth inequality, less polygyny: Rethinking the polygyny threshold model. *Journal of The Royal Society Interface* **15**, 20180035 (2018).

[9] Cobb, C. W. & Douglas, P. H. A theory of production. *The American Economic Review* **18**, 139–165 (1928).

[10] Oh, S.-Y., Ross, C., Borgerhoff Mulder, M. & Bowles, S. The decline of polygyny: An interpretation. *Santa Fe Institute Working Paper* (2017). URL https://sfi-edu.s3.amazonaws.com/sfi-edu/production/uploads/working_paper/pdf/2017-12-037_ae5724.pdf.

[11] Spencer, P. Polygyny as a measure of social differentiation in Africa. In Mitchell, J. (ed.) *Numerical Techniques in Social Anthropology* (Philadelphia: Institute for the Study of Human Issues, 1980).

[12] Hoffman, M. D. & Gelman, A. The No-U-Turn sampler: Adaptively setting path lengths in Hamiltonian Monte Carlo. *Journal of Machine Learning Research* (2014).

[13] Stan Development Team. Stan: A C++ library for probability and sampling, version 2.8.0 (2015). URL http://mc-stan.org/.

[14] R Core Team. *R: A Language and Environment for Statistical Computing*. R Foundation for Statistical Computing, Vienna, Austria (2015). URL https://www.R-project.org/.

[15] Courtiol, A., Pettay, J. E., Jokela, M., Rotkirch, A. & Lummaa, V. Natural and sexual selection in a monogamous historical human population. *Proceedings of the National Academy of Sciences* **109**, 8044–8049 (2012). URL http://www.pnas.org/content/109/21/8044.abstract. http://www.pnas.org/content/109/21/8044.full.pdf.

[16] Fieder, M. & Huber, S. The effects of sex and childlessness on the association between status and reproductive output in modern society. *Evolution and Human Behavior* **28**, 392–398 (2007).

[17] Nettle, D. & Pollet, T. V. Natural selection on male wealth in humans. *The American Naturalist* **172**, 658–666 (2008).

[18] Filliben, J. J. The probability plot correlation coefficient test for normality. *Technometrics* **17**, 111–117 (1975).

Figure S1: Scatter plots of adult fertility rate on year of birth.

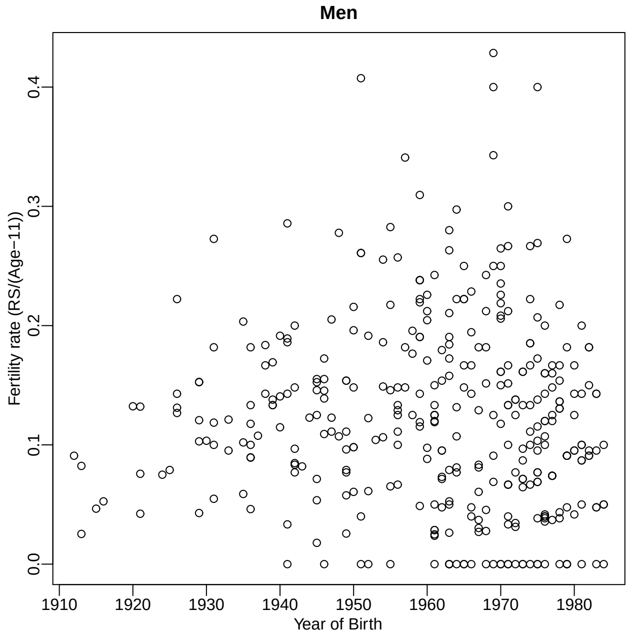

(a) Males

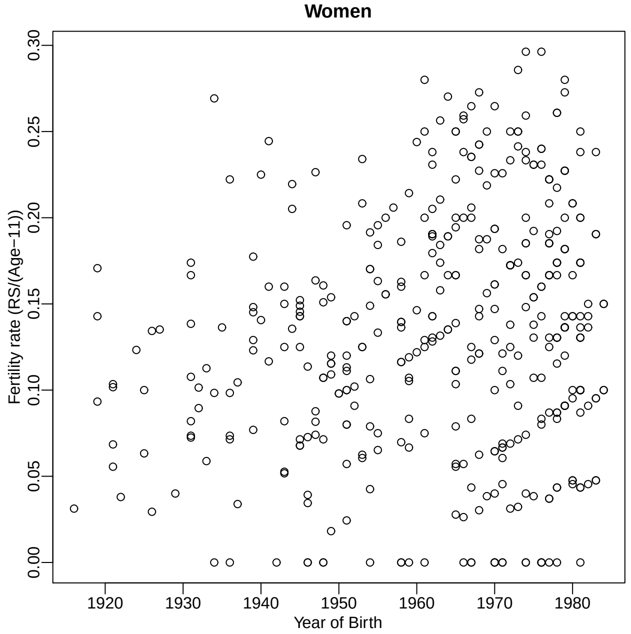

(b) Females

Figure S2: Frequency of polygyny in the Pimbwe over time. The precipitous decline in sample size in 2012 and 2014 reflect incomplete censuses of the village in those two years.

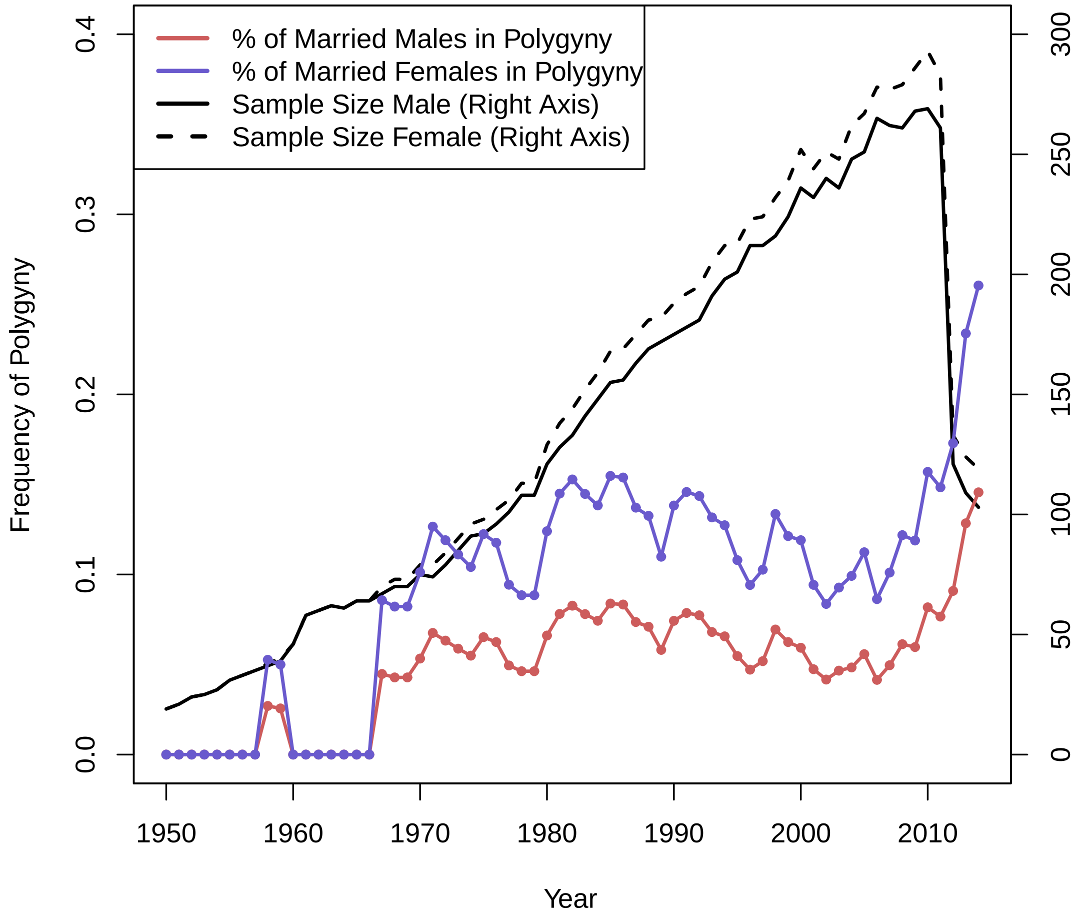

Figure S3: Bivariate scatters and correlations. Subset of individuals with > 0 unique spouses.

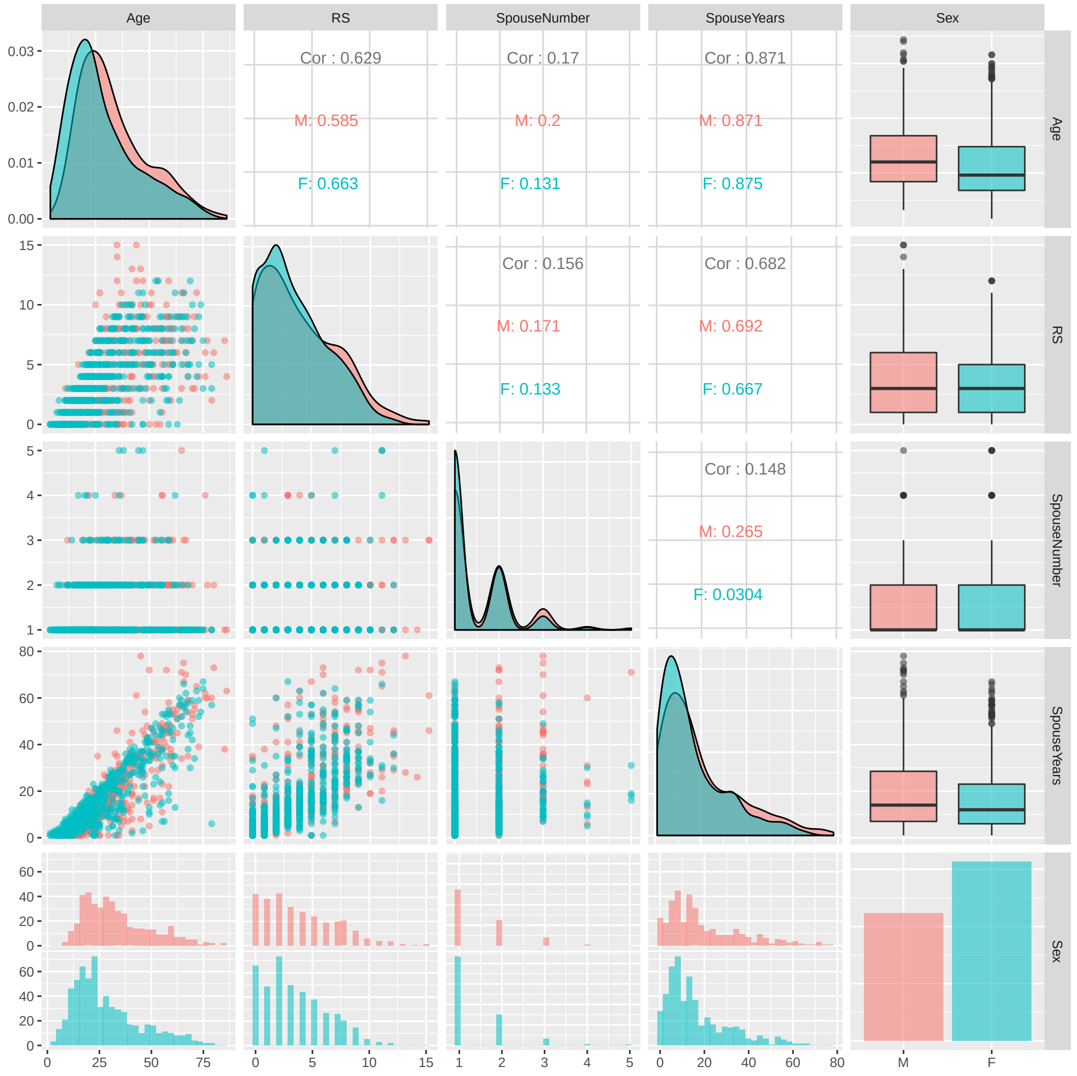

Figure S4: Bivariate scatters and correlations. Subset of individuals with ≥ 45 years of age.

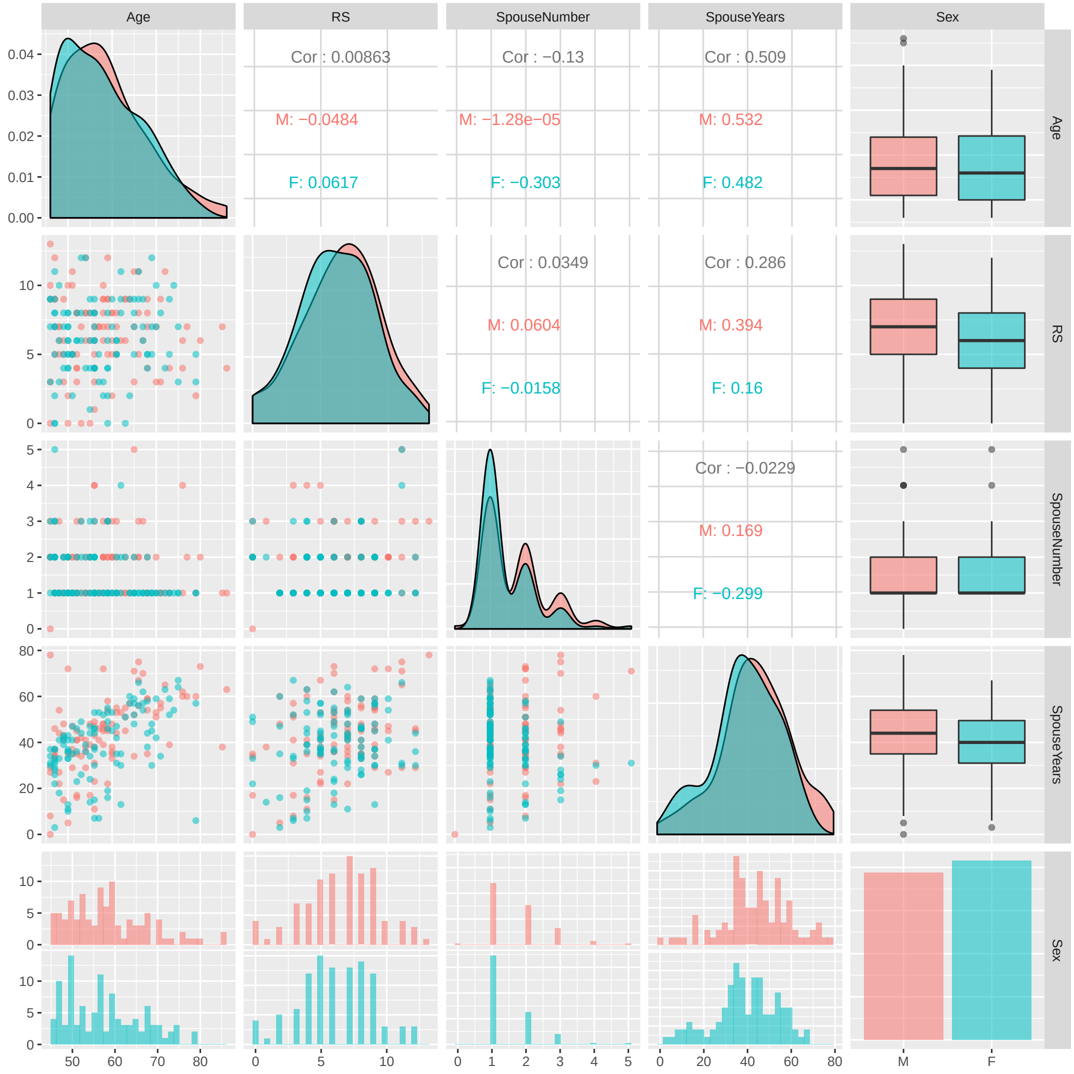

Figure S5: Bivariate scatters and correlations. Subset of individuals with > 1 unique spouse.

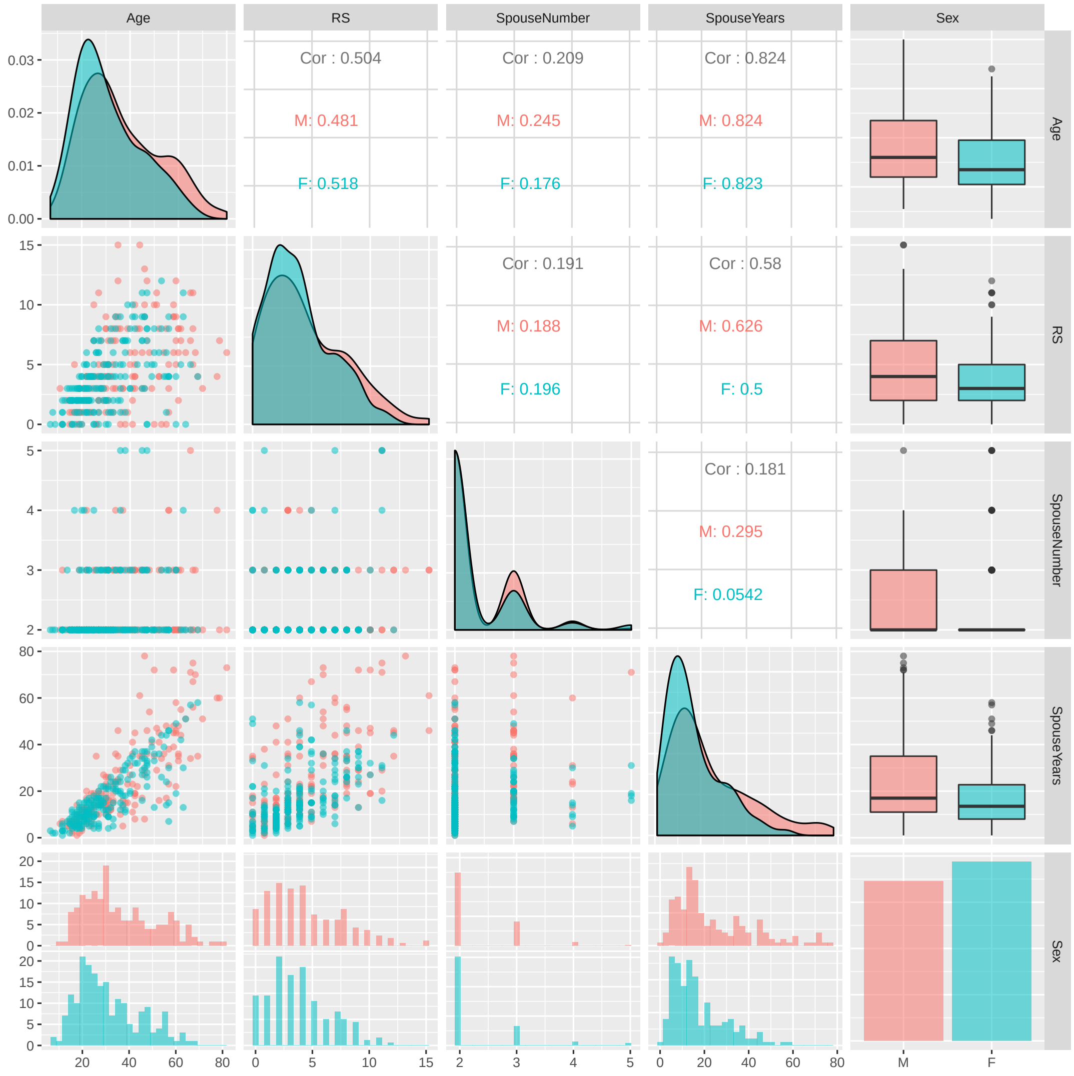

Figure S6: Bivariate scatters and correlations. Full dataset.

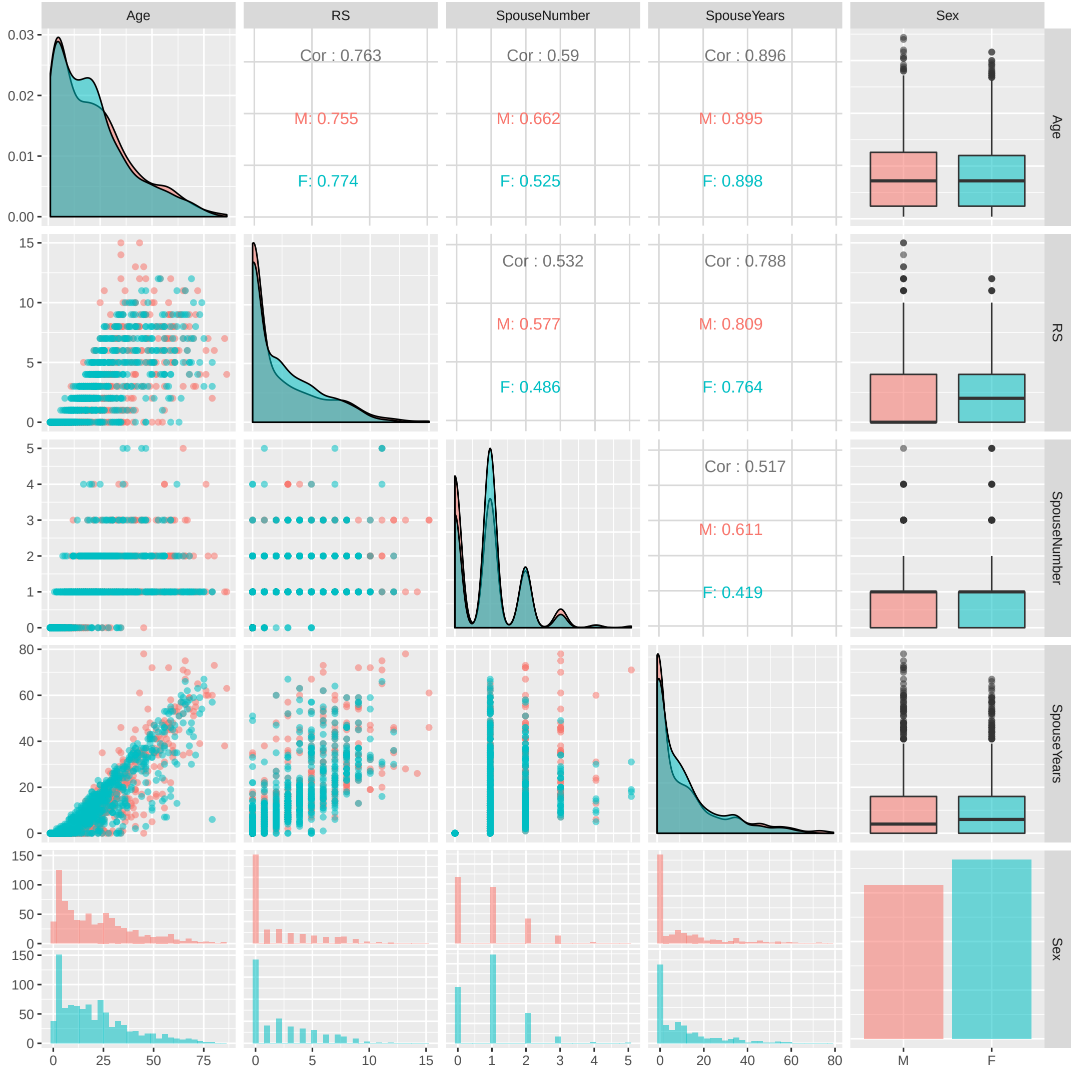

Figure S7: Sex-specific slope estimates of RS on years married calculated from regression parameters in the 'marital years model' using the full dataset as presented in the main text. Estimates, nevertheless, are predicted value for males and females at age 45. Note that the *slope* depends on years married if the *elasticity* on years married is not equal to one. The dashed vertical bars show the 90% density intervals of the data for years married for the subset of individuals between ages 44 and 46. The model predictions are best considered only in this range. We see that the male slope estimate is higher than the corresponding female slope estimate over almost the entire data range—a result concordant with our findings as presented in the main paper.

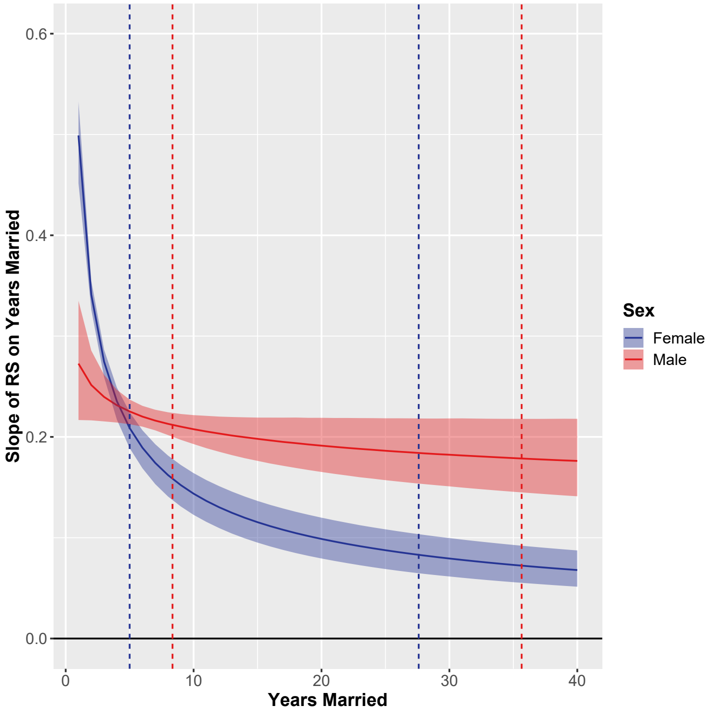

Figure S8: Sex-specific slope estimates of RS on years married calculated from regression parameters in the 'marital years model' as presented in section 4.2 of this supplement. Estimates are predicted values for males and females at age 45. Note that the *slope* depends on years married if the *elasticity* on years married is not equal to one. The dashed vertical bars show the 90% density intervals of the data for years married for the subset of individuals between ages 45 and 46. The model predictions are best considered only in this range. We see that the male slope estimate is higher than the corresponding female slope estimate over almost the entire data range—a result concordant with our findings as presented in the main paper.

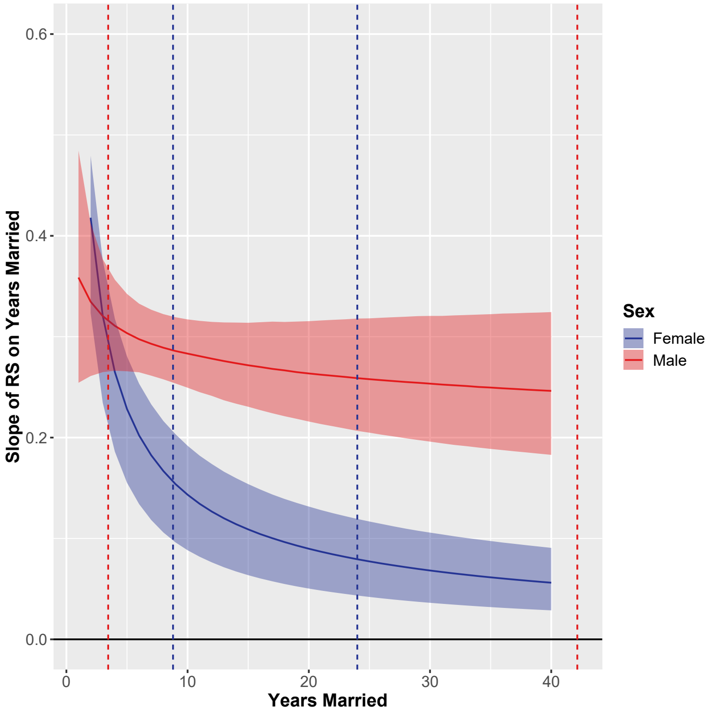

Figure S9: Histogram of male (red) and female (blue) RS, matched to the counter-factual histogram if the same number of observations were drawn from a Poisson distribution with the same sex-specific mean (dark grey). Note that our data are highly non-Gaussian. We observe a large peak at $RS = 0$, due mostly to young individuals who have never been married. We deal with this density at $RS = 0$, by using a variant of a standard zero-inflated model. Note also that RS is over-dispersed relative to a Poisson distribution (this means that the data distribution has higher density in both tail regions than the standard Poisson distribution). We deal with this by using a negative binomial regression framework.

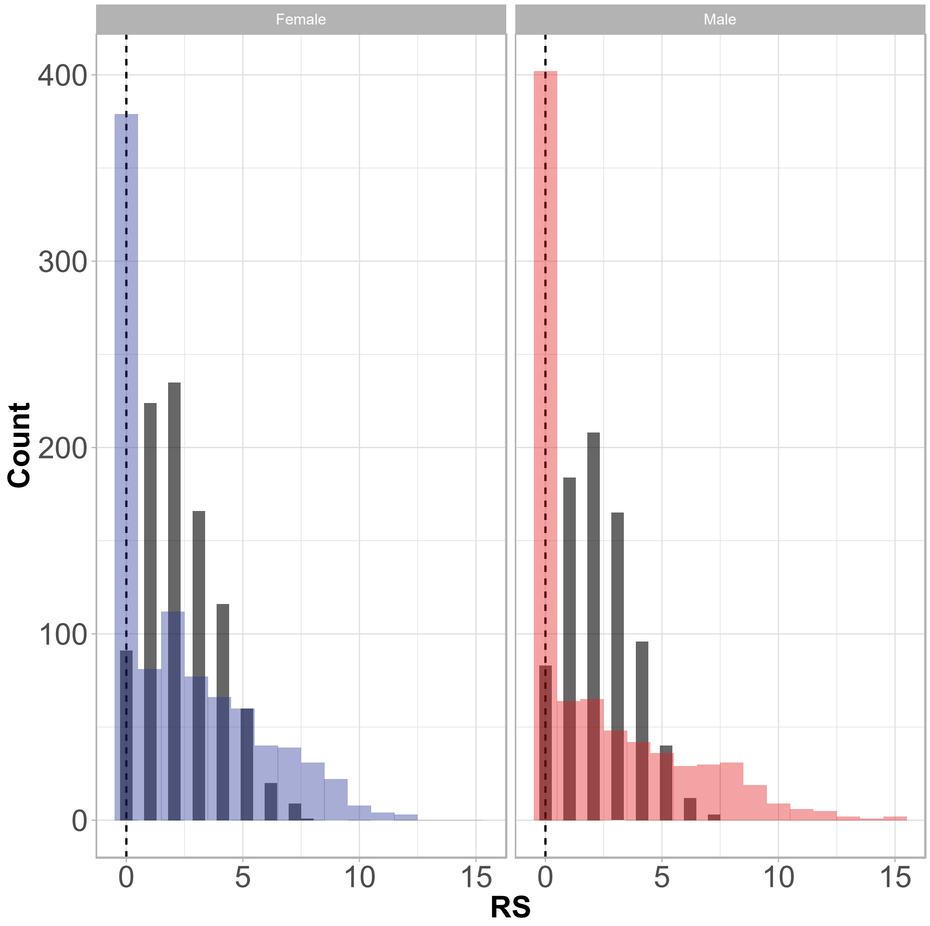

Figure S10: A schematic representation of the full model used in analysis.

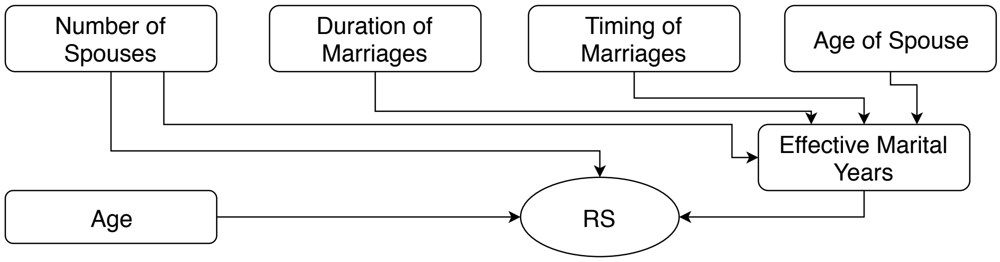

Figure S11: Traceplots of the main model parameters, β. For each model, both chains (colored arbitrarily) appear to have converged to the same posterior region and to have mixed thoroughly.

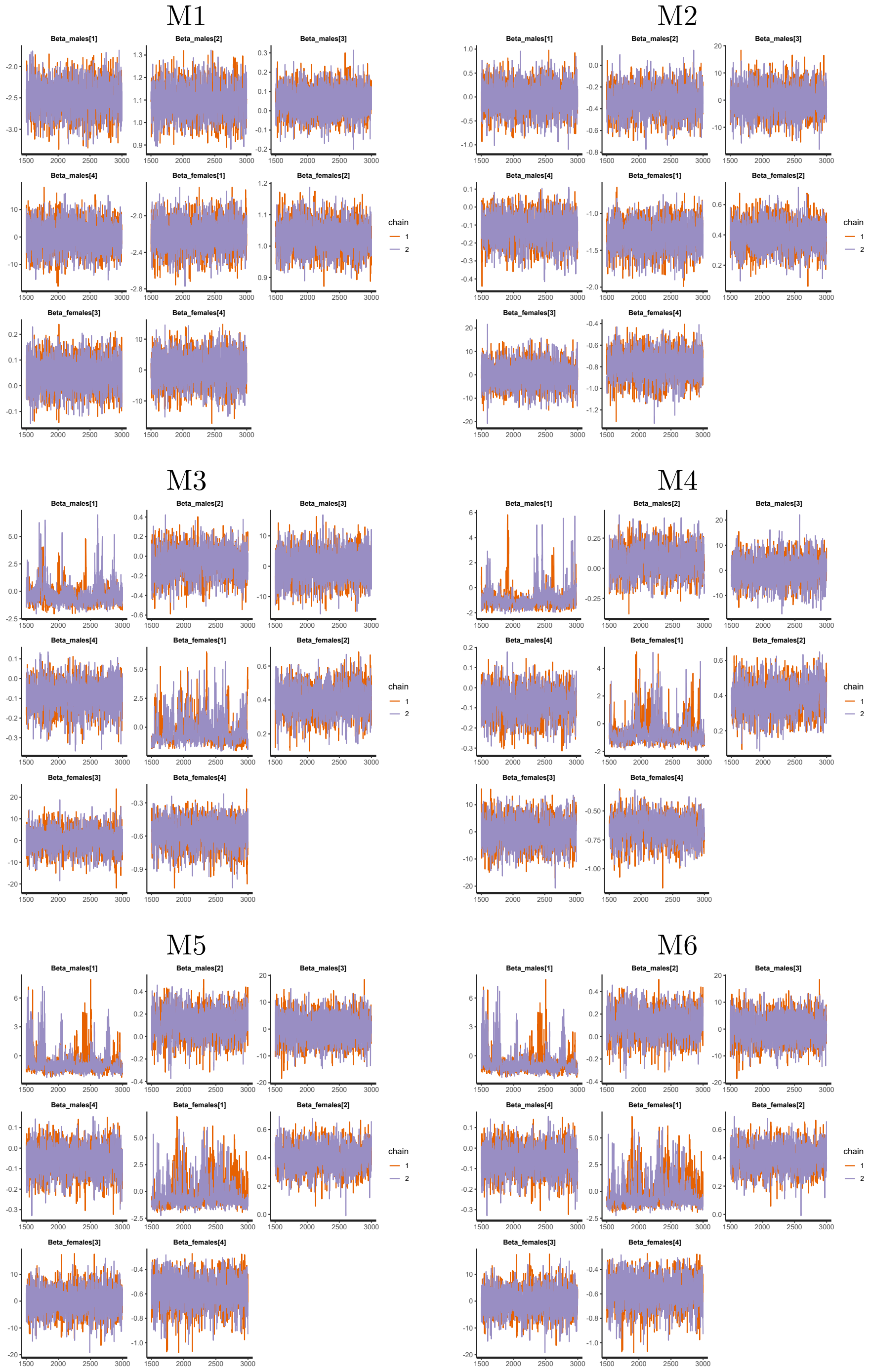

Figure S12: Sex-specific probability of remaining never-married by a given age. Dark lines plot medians and the shaded areas plot the 90% posterior density intervals of the mean.

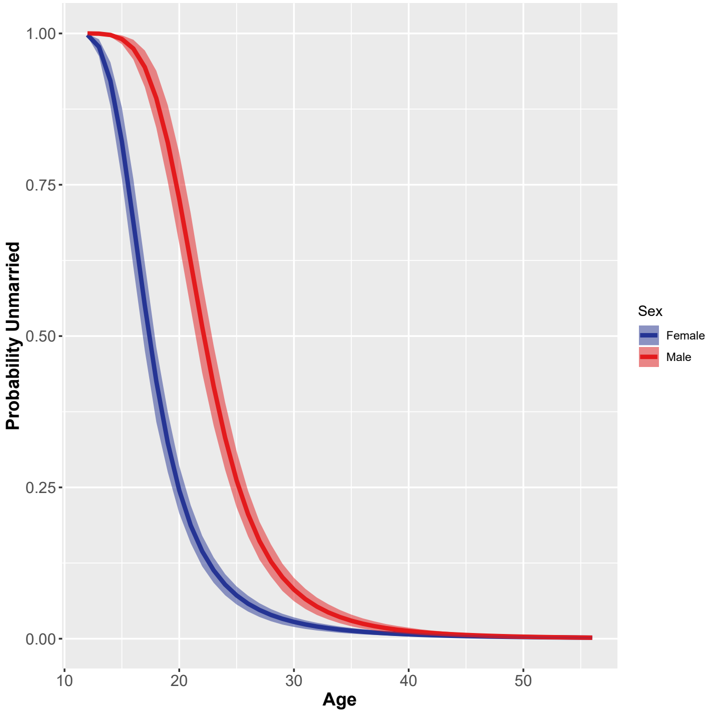

Figure S13: Reproductive success and age in never-married individuals. Dark lines plot medians and the shaded areas plot the 90% posterior density intervals of the mean.

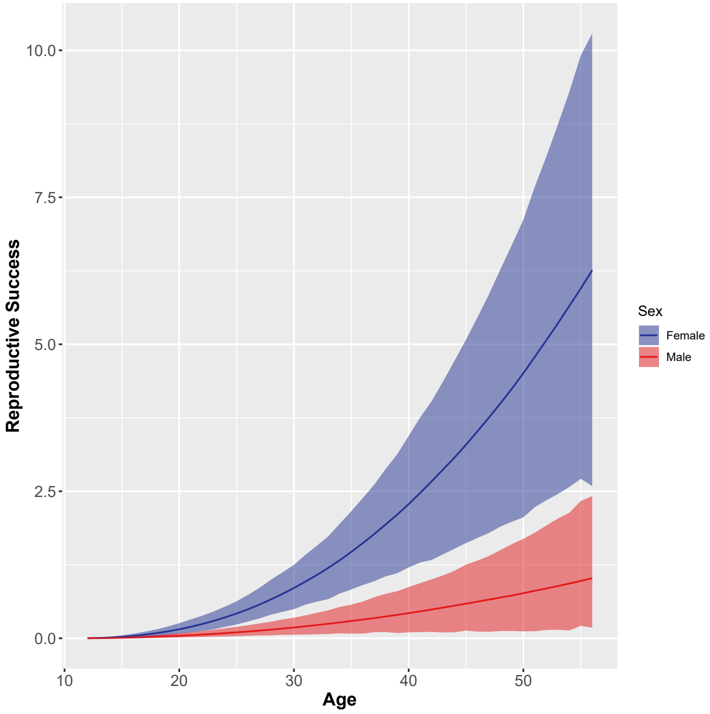

Figure S14: Density of RS among never-married individuals.

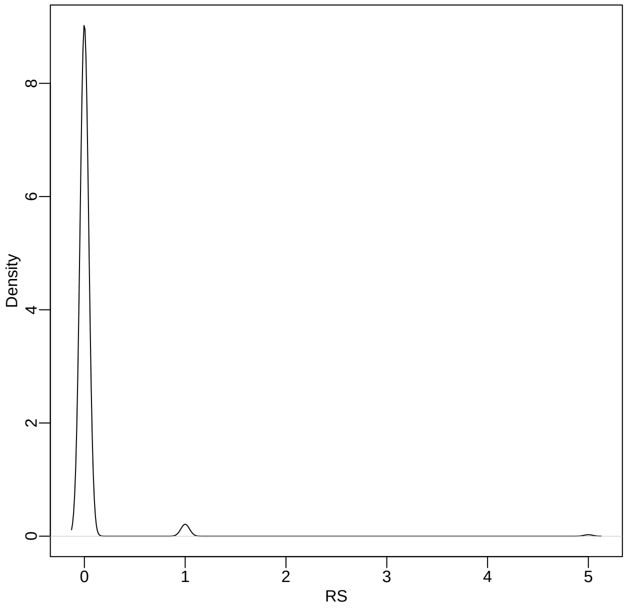

(a) Males

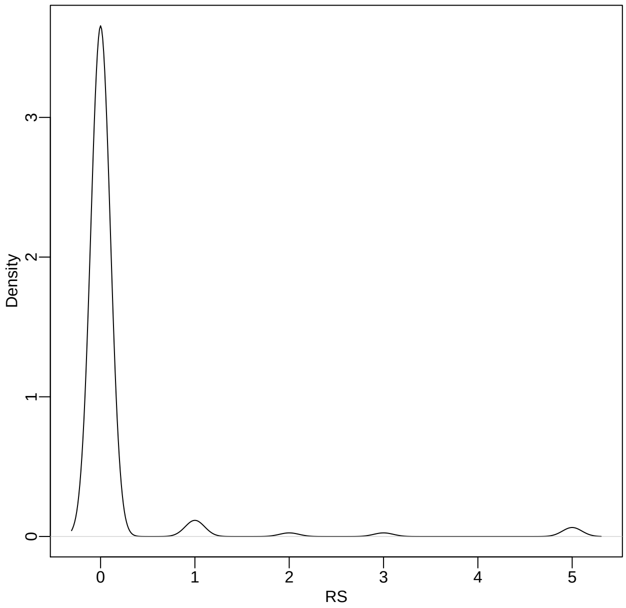

(b) Females

Figure S15: Relative value of having a spouse *at* a given age (marriage timing weight) from the model with only marriage timing weights. The shaded regions plot smoothed highest 65% posterior density intervals, and the solid lines plot the smoothed posterior medians.

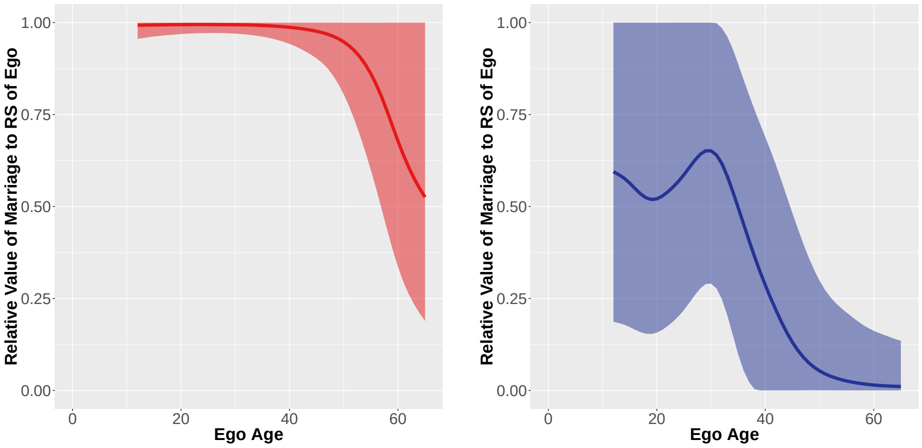

(a) Focal males (b) Focal females

Figure S16: Relative value of having a spouse *of* a given age (spousal quality weight) from the model with only spousal quality weights. The shaded regions plot smoothed highest 65% posterior density intervals, and the solid lines plot the smoothed posterior medians.

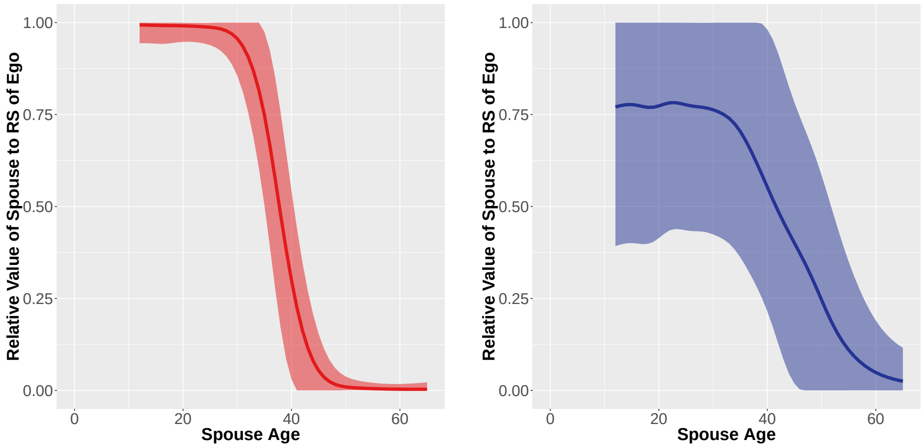

(a) Focal males

(b) Focal females

Figure S17: Q-Q or Quantile-Quantile plot of the standardized residuals from the full OLS model using the full dataset. Under the assumption that the model residuals are normally distributed—as assumed by the regression model—the plotted points should lie on the diagonal grey line. Note, however, the substantial divergence from the theoretical expectations. The steeper slope of the plotted points suggest general over-dispersion relative to a normal distribution, and the flat middle region suggests a spike of identical values. For both males and females, the model assumptions of normally-distributed residual are severely violated.

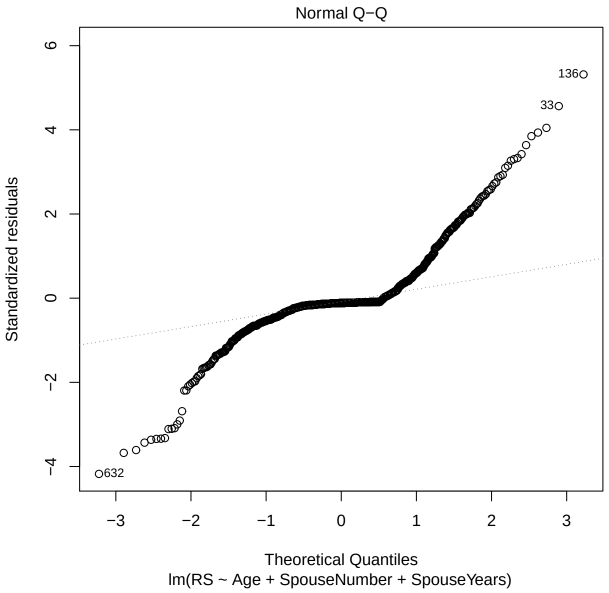

(a) Males

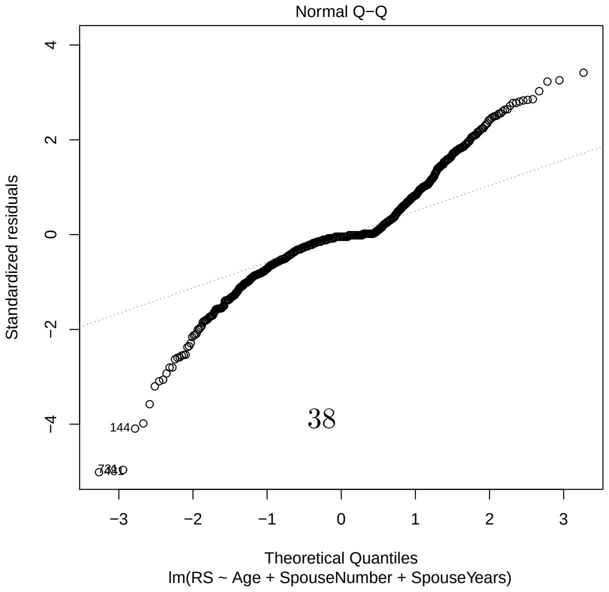

(b) Females

Figure S18: Q-Q or Quantile-Quantile plot of the standardized residuals from the full OLS model using the dataset that includes only individuals of age 45 and over. Under the assumption that the model residuals are normally distributed—as assumed by the regression model—the plotted points should lie on the diagonal grey line. This is indeed what we find here.

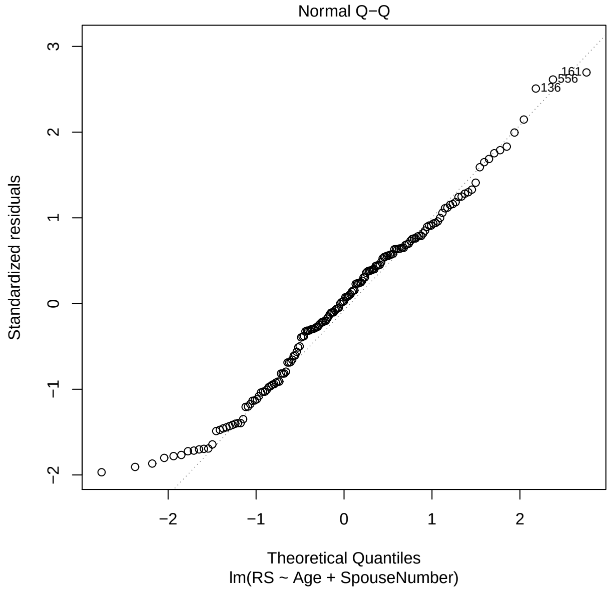

(a) Males

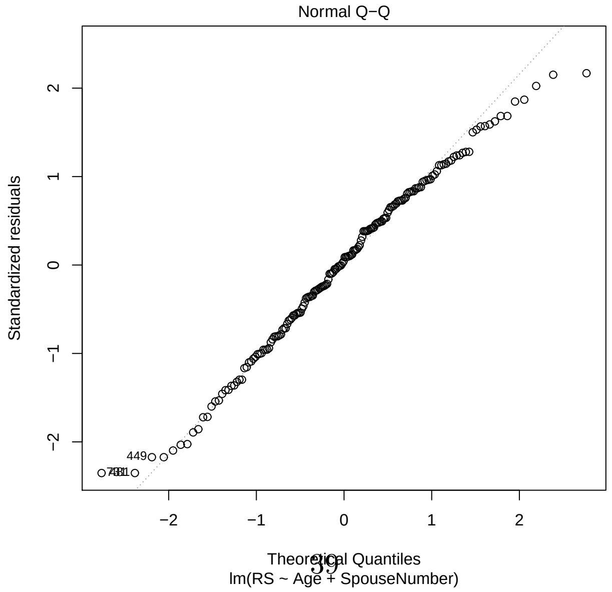

(b) Females

Table S1: Adult (age $\geq$ 30) rate of RS production as a function of year of birth

	Dependent variable:	
	Male Fertility rate, $\frac{RS}{Age-11}$	Female Fertility rate, $\frac{RS}{Age-11}$
	(1)	(2)
Year of Birth	-0.0003	0.001^{***}
	(0.0003)	(0.0002)
Constant	0.628	-1.481^{***}
	(0.500)	(0.420)
Observations	370	421
R^2	0.003	0.034
Adjusted R^2	0.0001	0.032
Residual Std. Error	0.080 (df = 368)	0.070 (df = 419)
F Statistic	1.021 (df = 1; 368)	14.785^{***} (df = 1; 419)
Note:		*p<0.1; **p<0.05; ***p<0.01

Table S2: Male-to-female contrasts under various definitions of marriage success for individuals older than age 55. The column labeled *Principle 2, MS* provides the male-to-female contrast in inequality in marriage success. The symbol I_s refers to the opportunity for sexual selection. For the opportunity for sexual selection metric, the contrast $\delta(\cdot)$ indicates the difference in the log of the male and female values—e.g., $\log(I_{s_m}) - \log(I_{s_f})$. Confidence intervals on the opportunity for sexual selection metric are bootstrapped. All confidence intervals are 90 percent intervals—$n_m = 97$ and $n_f = 104$.

	**Principle 2, MS**
**Model**	δ ($\mathbf{I_s}$)
**Spouse Number**	0.03 (-0.33, 0.41)
**Marital Years**	-0.06 (-0.49, 0.33)
**Marital Years: Timing Weights**	0.58 (-0.04, 1.12)
**Marital Years: Spousal Age Weights**	0.15 (-0.35, 0.59)
**Marital Years: Both Weights**	0.57 (0.01, 1.17)
**Full Model**	0.56 (0.02, 1.16)

Table S3: Bateman analysis under various definitions of marriage success for post-reproductive men and women (of age 45 and over). The section labeled *Principle 2, MS* provides the opportunity for sexual selection metric, I_s, for the various included definitions of marriage success for each sex. The data used to calculate all measures come from individuals of age 45 or over ($n_m = 171$, $n_f = 176$). In the section labeled *Principle 3, Bateman Gradient*, we present the estimated regression coefficients: an intercept and the elasticities on age, spouse number (N), and—sometimes weighted—marital years, (Y), for males and females in each of the six statistical models. Each row presents the results of an independent model using a different measure, or combination of measures, of marriage success. A blank cell in the table indicates that the corresponding variable was not included in the model presented on that row. The section labeled *WAIC* contains the WAIC scores used for model comparison. Model comparisons are specific to sex. The term *WAIC* gives the WAIC information criteria, the term Δ gives the WAIC difference relative to the best model in the set, and ω gives the corresponding WAIC weight. Intervals on the opportunity for sexual selection metrics are bootstrapped confidence intervals; otherwise, the values reflect posterior credible intervals. All intervals are 90 percent intervals.

**Model**		**Principle 2, MS**	**Principle 3, Bateman Gradient**				**WAIC**		
		**I_s**	**Intercept**	**Age**	**N**	**Y**	**WAIC**	**Δ**	**ω**
**Spouse Number**	M	0.28 (0.24, 0.32)	0.19 (-0.96, 1.46)	0.43 (0.12, 0.74)	-0.14 (-0.31, 0.03)		891	113.09	0
	F	0.32 (0.25, 0.39)	0.86 (-0.21, 1.88)	0.25 (-0.04, 0.49)	-0.07 (-0.21, 0.08)		868	32.97	0
**Marital Years**	M	0.25 (0.2, 0.29)	2.92 (1.93, 3.91)	-1.09 (-1.42, -0.79)		0.9 (0.74, 1.04)	804.61	26.7	0
	F	0.19 (0.15, 0.22)	1.29 (0.33, 2.42)	-0.16 (-0.47, 0.17)		0.33 (0.18, 0.46)	848.18	13.15	0
**Marital Years: Marriage Timing Weights**	M	0.21 (0.15, 0.27)	1.92 (0.03, 3.59)	-0.77 (-1.29, -0.26)		0.9 (0.76, 1.05)	800.95	23.04	0
	F	0.1 (0.06, 0.13)	0.55 (-0.52, 1.6)	-0.05 (-0.33, 0.2)		0.51 (0.32, 0.7)	835.03	0	0.6
**Marital Years: Spousal Quality Weights**	M	0.19 (0.15, 0.24)	1.14 (-0.07, 2.34)	-0.57 (-0.9, -0.21)		0.95 (0.81, 1.1)	784	6.09	0.04
	F	0.16 (0.12, 0.21)	1.21 (0.22, 2.27)	-0.15 (-0.45, 0.13)		0.38 (0.23, 0.53)	844.52	9.49	0.01
**Marital Years: Both Weights**	M	0.19 (0.14, 0.23)	0.76 (-0.44, 2.02)	-0.45 (-0.8, -0.11)		0.96 (0.8, 1.1)	780.47	2.56	0.21
	F	0.1 (0.07, 0.15)	0.67 (-0.47, 1.73)	-0.05 (-0.32, 0.25)		0.49 (0.3, 0.68)	836.24	1.21	0.33
**Full Model**	M	0.19 (0.14, 0.23)	0.73 (-0.7, 1.83)	-0.47 (-0.79, -0.13)	-0.17 (-0.3, -0.06)	1.01 (0.86, 1.15)	777.91	0	0.75
	F	0.1 (0.07, 0.15)	0.62 (-0.67, 1.78)	-0.04 (-0.33, 0.23)	0 (-0.14, 0.13)	0.48 (0.3, 0.7)	839.44	4.41	0.07

Table S4: Male-to-female contrasts under various definitions of marriage success for individuals of age 45 and older ($n_m = 171$, $n_f = 176$). The section labeled *Principle 2, MS* provides the male-to-female contrast in inequality in marriage success. The section labeled *Principle 3, Bateman Gradient* provides the male-to-female contrast in the effects of spouse number and—sometimes weighted—marital years on RS. The symbol I_s refers to the opportunity for sexual selection. For the opportunity for sexual selection metric, the contrast $\delta(\cdot)$ indicates the difference in the log of the male and female values—e.g., $\log(I_{s_m}) - \log(I_{s_f})$; for the regression parameters on spouse number (N) and marital years (Y), it equals the difference of male and female values—e.g., $\beta_{N_m} - \beta_{N_f}$. Each row presents the results of an independent model using a different measure, or combination of measures, of marriage success. A blank cell in the table indicates that the corresponding variable was not included in the model presented on that row.

**Model**	**Principle 2, MS**	**Principle 3, Bateman Gradient**	
	$\delta(I_s)$	$\delta(N)$	$\delta(Y)$
**Spouse Number**	-0.16 (-0.45, 0.09)	-0.07 (-0.3, 0.13)	
**Marital Years**	0.26 (-0.01, 0.54)		0.58 (0.39, 0.8)
**Marital Years: Marriage Timing Weights**	0.78 (0.32, 1.24)		0.39 (0.15, 0.64)
**Marital Years: Spousal Quality Weights**	0.16 (-0.22, 0.52)		0.57 (0.36, 0.79)
**Marital Years: Both Weights**	0.59 (0.14, 1.06)		0.47 (0.23, 0.72)
**Full Model**	0.61 (0.12, 1.04)	-0.17 (-0.36, 0)	0.52 (0.28, 0.76)

Table S5: Robustness checks: Ever Married. The first sub-table provides OLS estimates for males. The second sub-table provides the corresponding estimates for females. In both cases, model (1) contains only spouse number, model (2) contains only marital years, and model (3) contains both predictors.

	Dependent variable:		
	Male RS		
	(1)	(2)	(3)
Age	0.117***	−0.015	−0.015
	(0.008)	(0.014)	(0.014)
Spouse Number	0.247		−0.072
	(0.173)		(0.157)
Marital Years		0.146***	0.147***
		(0.013)	(0.014)
Constant	−0.335	1.587***	1.687***
	(0.357)	(0.299)	(0.370)
Observations	447	447	447
R^2	0.346	0.480	0.480
Adjusted R^2	0.343	0.478	0.477
Residual Std. Error	2.629 (df = 444)	2.343 (df = 444)	2.345 (df = 443)
F Statistic	117.231*** (df = 2; 444)	204.875*** (df = 2; 444)	136.412*** (df = 3; 443)
Note:			*p<0.1; **p<0.05; ***p<0.01

	Dependent variable:		
	Female RS		
	(1)	(2)	(3)
Age	0.114***	0.058***	0.052***
	(0.005)	(0.010)	(0.011)
Spouse Number	0.189		0.327***
	(0.122)		(0.119)
Marital Years		0.073***	0.079***
		(0.012)	(0.012)
Constant	−0.010	0.627***	0.252
	(0.227)	(0.177)	(0.223)
Observations	627	627	627
R^2	0.442	0.472	0.478
Adjusted R^2	0.440	0.470	0.476
Residual Std. Error	2.089 (df = 624)	2.032 (df = 624)	2.021 (df = 623)
F Statistic	246.991*** (df = 2; 624)	279.117*** (df = 2; 624)	190.518*** (df = 3; 623)
Note:			*p<0.1; **p<0.05; ***p<0.01

Table S6: Robustness checks: Individuals of age 45 and over. The first sub-table provides OLS estimates for males. The second sub-table provides the corresponding estimates for females. In both cases, model (1) contains only spouse number, model (2) contains only marital years, and model (3) contains both predictors.

	Dependent variable:		
	Male RS		
	(1)	(2)	(3)
Age	0.026	−0.133***	−0.134***
	(0.022)	(0.025)	(0.025)
Spouse Number	0.218		−0.222
	(0.297)		(0.247)
Marital Years		0.157***	0.160***
		(0.017)	(0.017)
Constant	4.523***	7.366***	7.714***
	(1.183)	(0.949)	(1.026)
Observations	171	171	171
R^2	0.013	0.342	0.345
Adjusted R^2	0.001	0.334	0.334
Residual Std. Error	3.361 (df = 168)	2.743 (df = 168)	2.745 (df = 167)
F Statistic	1.063 (df = 2; 168)	43.682*** (df = 2; 168)	29.355*** (df = 3; 167)
Note:			*p<0.1; **p<0.05; ***p<0.01

	Dependent variable:		
	Female RS		
	(1)	(2)	(3)
Age	0.029	−0.022	−0.021
	(0.019)	(0.023)	(0.023)
Spouse Number	0.075		0.212
	(0.239)		(0.235)
Marital Years		0.061***	0.064***
		(0.018)	(0.018)
Constant	4.438***	5.068***	4.589***
	(1.096)	(0.920)	(1.062)
Observations	176	176	176
R^2	0.014	0.076	0.080
Adjusted R^2	0.002	0.065	0.064
Residual Std. Error	2.746 (df = 173)	2.658 (df = 173)	2.659 (df = 172)
F Statistic	1.187 (df = 2; 173)	7.083*** (df = 2; 173)	4.990*** (df = 3; 172)
Note:			*p<0.1; **p<0.05; ***p<0.01

Table S7: Robustness checks: All data. The first sub-table provides OLS estimates for males. The second sub-table provides the corresponding estimates for females. In both cases, model (1) contains only spouse number, model (2) contains only marital years, and model (3) contains both predictors.

	Dependent variable:		
	Male RS		
	(1)	(2)	(3)
Age	0.113***	0.026***	0.015*
	(0.005)	(0.008)	(0.008)
Spouse Number	0.462***		0.389***
	(0.104)		(0.093)
Marital Years		0.131***	0.129***
		(0.009)	(0.009)
Constant	−0.611***	0.215*	0.147
	(0.113)	(0.114)	(0.113)
Observations	791	791	791
R^2	0.581	0.660	0.667
Adjusted R^2	0.579	0.659	0.666
Residual Std. Error	2.026 (df = 788)	1.825 (df = 788)	1.806 (df = 787)
F Statistic	545.255*** (df = 2; 788)	764.006*** (df = 2; 788)	526.037*** (df = 3; 787)
Note:			*p<0.1; **p<0.05; ***p<0.01

	Dependent variable:		
	Female RS		
	(1)	(2)	(3)
Age	0.116***	0.073***	0.055***
	(0.004)	(0.007)	(0.008)
Spouse Number	0.358***		0.452***
	(0.079)		(0.077)
Marital Years		0.072***	0.080***
		(0.009)	(0.009)
Constant	−0.368***	0.073	−0.087
	(0.097)	(0.097)	(0.099)
Observations	922	922	922
R^2	0.607	0.624	0.637
Adjusted R^2	0.606	0.623	0.636
Residual Std. Error	1.763 (df = 919)	1.726 (df = 919)	1.695 (df = 918)
F Statistic	710.127*** (df = 2; 919)	761.122*** (df = 2; 919)	537.366*** (df = 3; 918)
Note:			*p<0.1; **p<0.05; ***p<0.01
